# Efficacy and Side Effect Profile of Different Formulations of Metformin: A Systematic Review and Meta-Analysis

**DOI:** 10.1007/s13300-021-01058-2

**Published:** 2021-06-02

**Authors:** Jane L. Tarry-Adkins, Imogen D. Grant, Susan E. Ozanne, Rebecca M. Reynolds, Catherine E. Aiken

**Affiliations:** 1 Department of Obstetrics and Gynaecology, The Rosie Hospital and NIHR Cambridge Biomedical Research Centre, University of Cambridge, Cambridge, CB2 0SW UK; 2 Metabolic Research Laboratories and MRC Metabolic Diseases Unit, Wellcome Trust–MRC Institute of Metabolic Science, University of Cambridge, Cambridge, UK; 3 Centre for Cardiovascular Science, Queens Medical Research Institute, Edinburgh Bioquarter, 47 Little France Crescent, Edinburgh, EH16 4TJ UK

**Keywords:** Diabetes, Efficacy, Metformin, Polycystic ovarian syndrome, Side effects

## Abstract

**Introduction:**

Metformin is among the most frequently prescribed drugs worldwide for a variety of indications. Although metformin has several important advantages, for example being easy to store and administer, it is associated with a high incidence of gastrointestinal side effects. Slower-release formulations of metformin may reduce the incidence of side effects while maintaining efficacy; however, there is a lack of systematic evidence available to guide head-to-head comparisons between different metformin formulations.

**Methods:**

PubMed, Web of Science, OVID EMBASE, MEDLINE, The Cochrane database and Clinicaltrials.gov were systematically searched (from inception to 25 January 2021). Trials that randomized adult participants to extended-release formulation of metformin (met-XR), delayed-release (met-DR) or immediate-release metformin (met-IR) were included. Two reviewers independently assessed articles for eligibility and risk-of-bias, with conflicts resolved by a third reviewer. Outcome measures were change in fasting plasma glucose (FPG), glycated haemoglobin (HbA1c), body weight, BMI, lipid profile and side effects. Meta-analyses were conducted using random-effects models.

**Results:**

Fifteen studies (*n* = 3765) met eligibility criteria. There was no significant difference between the efficacy of met-IR, met-XR or met-DR in changing FPG (*p* = 0.93). A non-significant reduction in mean body weight was observed in individuals randomized to met-XR vs. met-IR (− 1.03 kg, 95% CI − 2.12 to 0.05, *p* = 0.06). Individuals randomized to met-XR vs. met-IR had lower low-density lipoprotein (LDL) cholesterol levels (− 5.73 mg/dl, 95% CI − 7.91 to − 3.56, *p* < 0.00001). Gastrointestinal (GI) side effects were markedly reduced in patients randomised to met-DR vs. met-IR (OR 0.45, 95% CI 0.26–0.80, *p* = 0.006).

**Conclusion:**

Our results demonstrate equal efficacy of longer-acting formulations (met-XR, met-DR) versus immediate-release metformin formulations in terms of glycaemic control. There were insufficient studies available to compare the efficacy of different metformin formulations outside of diabetes care. However met-XR was associated with reduced serum LDL cholesterol concentrations, while met-DR was strongly associated with reduced GI side effects, which could improve drug compliance.

**Supplementary Information:**

The online version contains supplementary material available at 10.1007/s13300-021-01058-2.

## Key Summary Points

**Table:** 

***Why carry out this study?***
Despite the very widespread clinical use of metformin, there is a lack of systematic evidence to guide optimal selection of the various formulations available.
***What was learned from the study?***
Long-acting metformin formulations (extended and delayed release) have equal efficacy in glycaemic control compared to immediate-release metformin
Metformin extended release is associated with reduced LDL cholesterol concentrations compared to immediate release.
Metformin delayed release was associated with reduced gastrointestinal side effects compared to immediate release, which could improve drug compliance.
Further research is required to refine the optimal cost–benefit ratio of the different available preparations of metformin in various clinical circumstances.

## Digital Features

This article is published with digital features, including a summary slide, to facilitate understanding of the article. To view digital features for this article go to https://doi.org/10.6084/m9.figshare.14355116.

## Introduction

Metformin (*N*,*N*-dimethylbiguanide) is increasingly prescribed worldwide for a widening variety of indications. In 2018, metformin was prescribed over 80 million times, making it the fourth most commonly prescribed drug in the USA [1]. Metformin is included in the World Health Organization (WHO) model list of essential medicines [2], reflecting its increasing use and suitability for low-resource settings. Despite the very widespread clinical use of metformin, there is a lack of systematic evidence to guide optimal selection of the various formulations available.

Metformin is primarily prescribed as an oral glucose-lowering agent in the context of type 2 diabetes mellitus (T2DM). Metformin is efficacious in controlling hyperglycaemia and thus minimising the long-term consequences of diabetes [3]. Metformin exerts glucose-lowering effects through several mechanisms including suppressing hepatic gluconeogenesis (via AMPK activation), downregulating lipogenic enzymes, and inhibiting cellular respiration (via inhibition of mitochondrial complex I) [4–7]. Metformin is also prescribed for other indications, including polycystic ovary syndrome (PCOS) [8], gestational diabetes [9] and obesity [10], although these uses are not licensed in the UK or USA. A mounting body of evidence also suggests that metformin may be of benefit in diverse conditions such as cancer treatment [11], dermatological conditions [12], pre-eclampsia [13] and osteoarthritis [14].

A significant barrier to the use of metformin is the high incidence of side effects, particularly gastrointestinal (GI) symptoms. Of all patients who take metformin, 20–30% report GI side effects; and approximately 5% of all patients discontinue treatment because of severe GI symptoms [15]. The concentration of metformin measured in the small intestine peaks at 30–300 times greater than plasma concentrations [16]; thus it has been suggested that intestinal accumulation of metformin may be a key driver of the reported GI side effects [17]. It is therefore plausible that different formulations of metformin, absorbed systemically at different rates and locations in the GI tract, may have significantly different GI side effect profiles. Metformin hydrochloride (metformin immediate release, met-IR), is poorly absorbed in the stomach [18], so the majority of the drug is absorbed in the upper part of the small intestine. Metformin extended release (met-XR) is a formulation of metformin hydrochloride suspended within a polymer matrix that dissolves over hours as the tablet passes through the GI tract. Peak metformin concentrations in the small intestine are thus reduced with met-XR compared to met-IR [19]. The newest formulation of metformin is metformin delayed release (met-DR) [19, 20]. Met-DR is distinct from met-XR in that it comprises a core of metformin hydrochloride with a pH-dependent enteric coating, which dissolves at pH ≥ 6.5. Hence drug delivery is targeted to the ileum, maximising the gut-based mechanisms of action of metformin while reducing bioavailability. Longer-acting formulations thus reduce systemic absorption of metformin [18, 19]. It is possible that lower doses of met-XR or met-DR could be used to achieve similar efficacy to met-IR while simultaneously reducing prevalence of side effects.

We systematically compared the efficacy and side effect profiles of met-IR, met-XR and met-DR. Despite the increased costs of longer-acting preparations of metformin, if there are significant gains in tolerability then increased use of these formulations may ultimately improve concordance, increase achievement of treatment goals and thus ultimately reduce spending within healthcare services.

## Methods

This systematic review and meta-analysis was conducted in accordance with the Preferred Reporting Items for Systematic Reviews and Meta-Analyses (PRISMA) guidelines [21]. The systematic review protocol was registered prospectively in PROSPERO (CRD42020167692; Supplementary Text S1) prior to data collection. This article is based on previously conducted studies and does not contain any new studies with human participants or animals performed by any of the authors and therefore ethical approval was not required.

### Literature Searches, Search Strategies and Eligibility Criteria

Systematic literature searches using pre-specified terms (Supplementary Text S2) were performed on PubMed, Ovid EMBASE, Ovid Medline, Cochrane library, Clinicaltrials.gov and Web of Science from database inception to 25 January 2021. No language or location restrictions were applied. Studies that randomised adults to any ‘extended-release’ formulation of metformin (encompassing slow-release, extended-release, controlled-release and delayed-release preparations) versus immediate-release metformin (met-IR) were included (Supplementary Table S1). Studies were included if they randomised patients for any indication. All treatment indications were screened for and diagnosed according to local criteria in each study, and we did not apply exclusions with respect to this. No restrictions were applied with respect to the length of follow-up period. Efficacy outcomes were change in fasting plasma glucose (FPG; mg/dl and mmol/l), change in glycated haemoglobin (HbA1c; %), body mass index (BMI; kg/m^2^), mean body weight (kg), lipid profiles (including total, HDL, LDL cholesterol and triglycerides; mg/dl). Any side effects, including GI effects, reported by individual studies were recorded. Data reported only in meeting abstracts was included if the abstract contained sufficient information for assessment. One meeting abstract [22] contained sufficient information and therefore was included in the meta-analysis. Where insufficient information for complete assessment was available, authors were contacted for further information. A total of seven authors were contacted; however none responded.

### Study Selection and Data Extraction

Two reviewers (JLT-A and IDG) independently assessed each study using pre-determined inclusion/exclusion criteria (Supplementary Table 1). A third reviewer (CEA) was available to resolve cases where eligibility was unclear. An initial screen of titles and abstracts was performed, followed by a detailed full paper screen (Supplementary Fig. S1). Data extraction from eligible studies was conducted independently using a standardized pro forma by two authors (JLT-A and IDG) with a third author (CEA) available if required.

### Quality Assessment (Risk of Bias)

Each study was independently assessed by two authors (JLT-A and IDG) for quality and validity using the Cochrane Collaboration tool for assessing risk of bias. A third reviewer (CEA) was available to resolve cases where risk of bias was unclear. Seven risk of bias domains were systematically assessed for each study and each domain was given a rating of low risk, unknown risk or high risk of bias (Supplementary Table S2). All risk of bias analysis was conducted at the study level.

### Statistical Analysis

The principal summary measures were unadjusted odds ratios (OR) (for dichotomous data) or mean difference (MD) (for continuous data). Meta-analysis and meta-regression were performed using Review Manager (RevMan Version 5.3, Copenhagen: The Nordic Cochrane Centre, the Cochrane Collaboration, 2014) and the metafor package in R version 3.5.1 (R Foundation for Statistical Computing, Vienna, Austria). Funnel plots were constructed to assess publication bias. Meta-analyses with five or more studies included were also subjected to Egger’s test. Heterogeneity between studies was assessed using the *I*^2^ statistic. We implemented random-effects meta-analyses using restricted maximum-likelihood (REML) estimator. Meta-regression was performed with fasting plasma glucose as the dependent variable and metformin dose as the predictor variable. Sensitivity analyses were performed using ‘leave-one-out’ (LOO) analysis for individual studies. All studies were analysed according to intention-to-treat. All outcomes were subjected to Grading of Recommendations, Assessment, Development and Evaluation (GRADE) analysis (GRADEpro Guideline Development Tool, McMaster University, USA). Where *p* values are reported, an alpha level < 0.05 was considered statistically significant.

## Results

### Study Selection and Study Characteristics

The PRISMA flow chart (Supplementary Fig. S1) demonstrates the screening procedure involved to include 15 eligible studies (*n* = 3765 participants). The majority of these studies (12 studies, involving 2934 participants) compared met-XR v. met-IR [22–33]. One study (*n* = 571) compared met-DR v. met-IR [20]. The remaining two studies (*n* = 260) compared met-DR, met-XR and met-IR [19, 34].

The majority of studies enrolled people with type 2 diabetes (13 studies; *n* = 3119). One study (*n* = 571) enrolled people with T2D and chronic kidney disease (CKD I/II) [20] and one study enrolled people with PCOS (*n* = 75) [22].

For all indications and comparisons, the studies varied with respect to quality and design (Supplementary Table S3). The included studies demonstrated heterogeneity with respect to the dosage of metformin (600–2500 mg) and the study duration (Supplementary Table S3). The included studies came from a variety of geographical locations: Europe (Belgium, Italy), Africa/Middle East (Pakistan, South Africa), Asia (China and Taiwan) and North America /Latin America (USA and Brazil).

### Risk of Bias and Sensitivity Analyses

The risk of bias was moderate-to-low in the majority of included studies; however two studies were open-label [27, 29]. Removal of these two studies did not substantively alter any outcomes, therefore all were included. We assessed the likelihood of single studies significantly influencing the overall results using LOO analysis (Supplementary Fig. S2) for met-IR v. met-XR comparisons. Two outcome measures investigated failed LOO analysis: change in body weight and likelihood of vomiting. This decreases our confidence of the robustness of these findings. Funnel plots for all outcomes were assessed visually (Supplementary Fig. S3); there were no obvious asymmetries in the plots for any study outcomes. All outcomes with more than five studies were subject to Egger’s test, and all passed.

#### GRADE Analysis (Certainty of Evidence)

The majority of outcomes were classified as having a moderate certainty of evidence (Supplementary Fig. S4), with one outcome having a high certainty of evidence (LDL cholesterol). The moderate certainty of evidence was due to from five outcomes having high heterogeneity between studies and four outcomes having high degrees of imprecision in the derived estimates. No publication bias (as ascertained by the Egger’s testing and funnel plot analyses) was detected. All studies reported direct evidence (Supplementary Fig. S4).

## Synthesis of Results

### Efficacy Outcomes

#### Mean Change in FPG

**Fig. 1:**
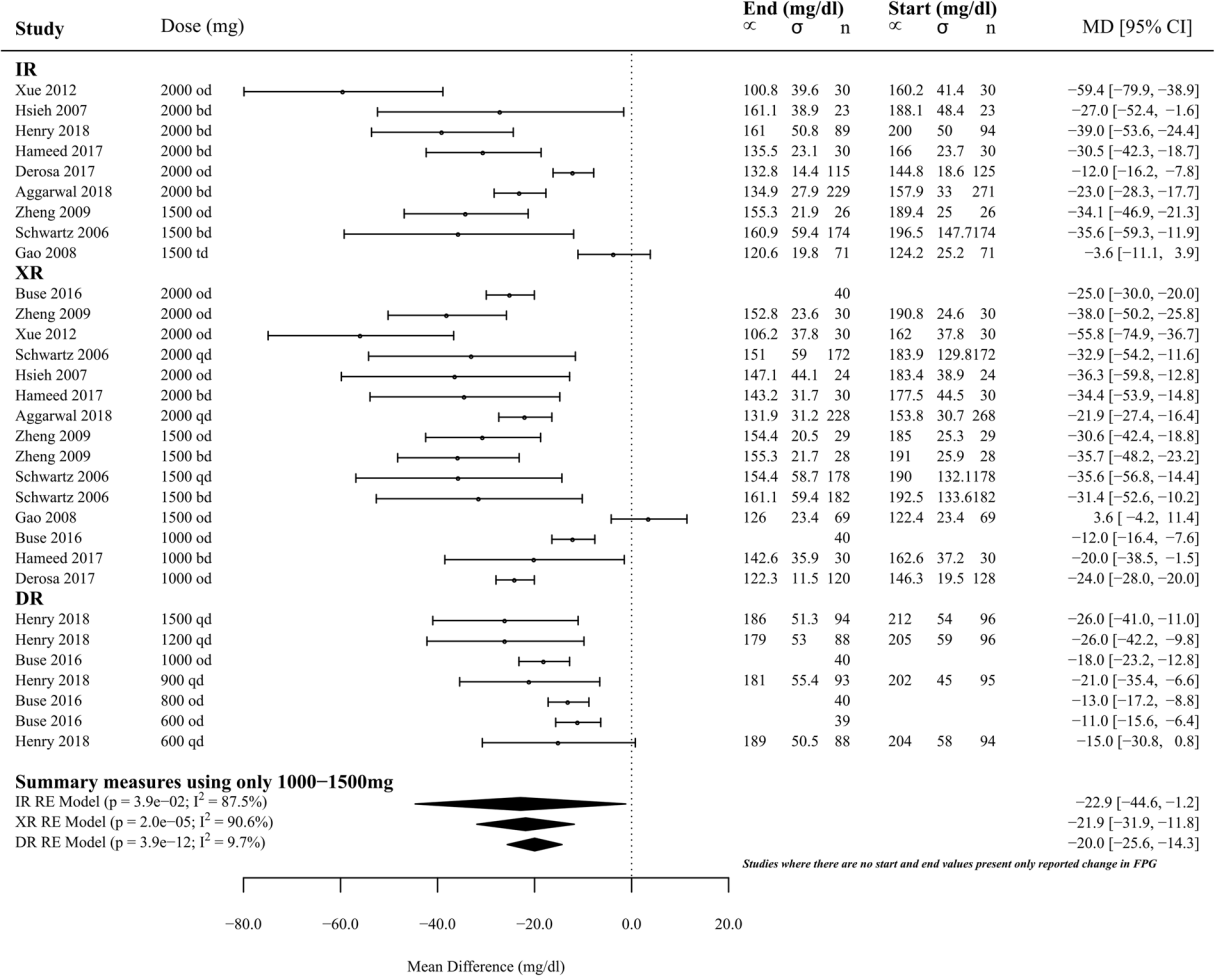
Effect of metformin formulations (met-XR, met-IR and met-DR) upon mean fasting plasma glucose concentrations (mg/dl) measured at the start and at the end of the study. Metformin doses range between 600 mg per day to 2000 mg. Summary measures expressed as doses between 1000 and 1500 mg per day. Mean difference and 95% confidence intervals

Ten studies (*n* = 2855) reported either change in FPG or start-and-finish FPG concentrations over 33 separate groups. Of these, two groups were intermediate measurements of the same participants, so 31 groups (*n* = 2499) were included in meta-analysis (9 met-IR, 15 met-XR, 7 met-DR). When summary measures were calculated using comparable dosages (1000–1500 mg), there was no significant difference between the efficacy of met-IR, met-XR or met-DR in changing FPG (*p* = 0.928, Fig. 1); met-IR, − 22.90 ± 21.70 mg/dl (1.30 ± 1.20 mmol/l): met-XR, − 21.90 ± 9.90 mg/dl (1.2 ± 0.6 mmol/l); met-DR, − 20.00 ± 5.60 mg/dl (1.10 ± 0.30 mmol/l).

**Fig. 2:**
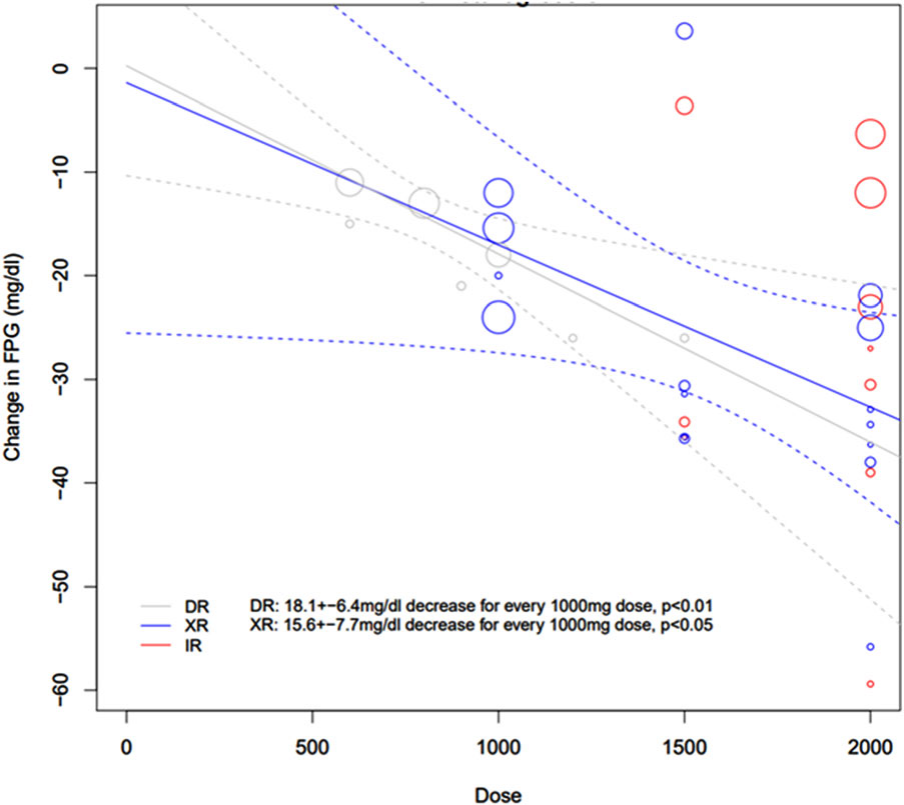
Meta-regression of the effect of metformin formulations (met-XR, met-IR and met-DR) upon the change in fasting plasma glucose concentrations

All 33 groups were included in meta-regression (10 met-IR, 16 met-XR, 7 met-DR, *n* = 2855). Met-IR was only reported at two doses, so meta-regression could not reasonably be performed. There was significant dose-dependency of change in FPG for both met-DR and met-XR. For every 1000 mg dose, FPG decreased by 0.90 ± 0.40 mmol/l (15.60 ± 7.70 mg/dl; *p* < 0.05) with met-XR and 1.00 ± 0.40 mmol/l (18.1 ± 6.4 mg/dl; *p* < 0.01) with met-XR (Fig. 2).

#### Mean Change in HbA1c

**Fig. 3:**
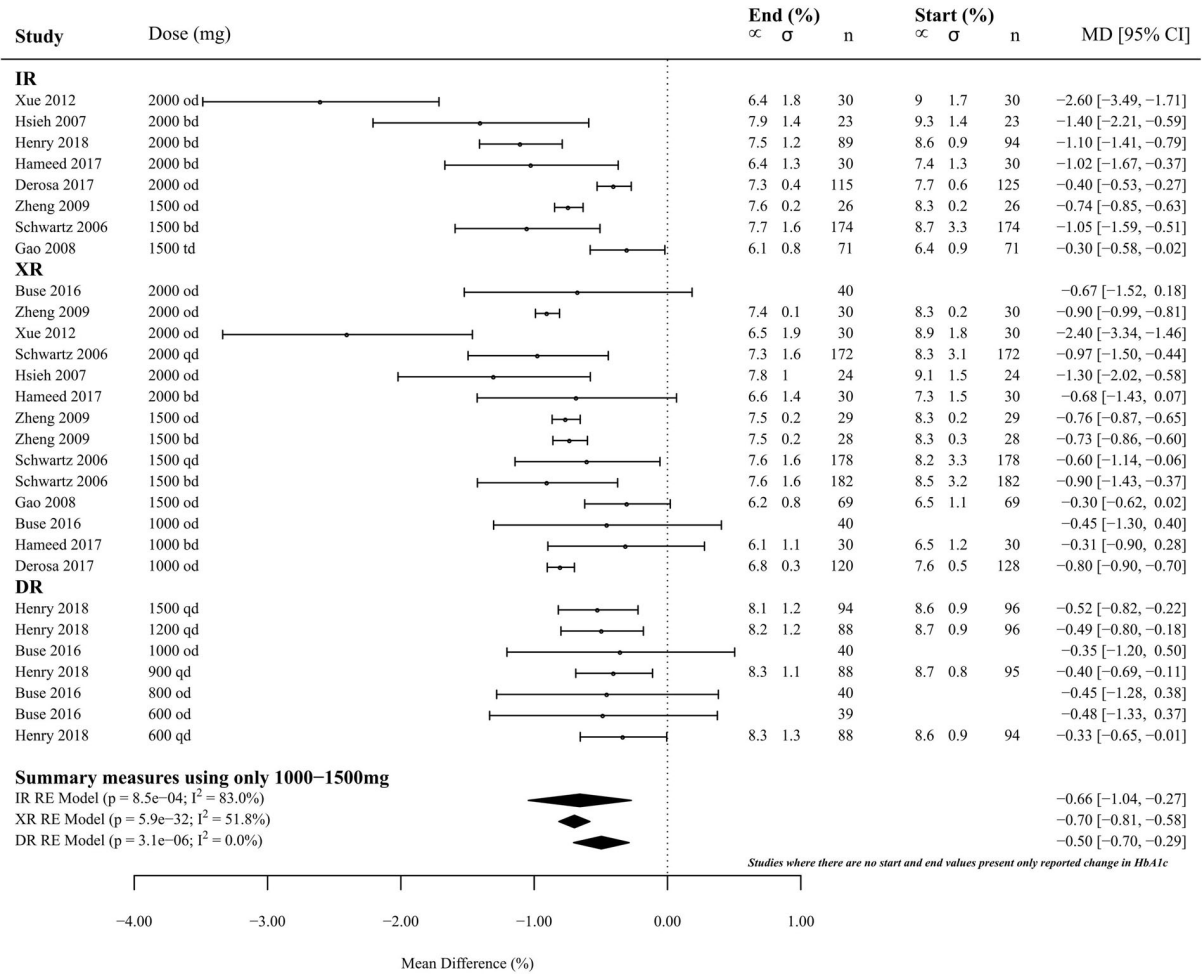
Effect of metformin formulations (met-XR, met-IR and met-DR) upon mean HbA1c concentrations (%) measured at the start and at the end of the study. Metformin doses range between 600 mg per day to 2000 mg. Summary measures expressed as doses between 1000 and 1500 mg per day. Mean difference and 95% confidence intervals

Nine studies (*n* = 2037) reported HbA1c change or start and finish HbA1c concentrations over 29 separate groups (8 met-IR, 14 met-XR, 7 met-DR). When summary measures were calculated using comparable dosages (1000–1500 mg), there was no significant difference between the efficacy of met-IR, met-XR or met-DR in reducing HbA1c (*p* = 0.25; met-IR, − 0.66 [− 1.04, − 0.27], *p* < 0.001; met-XR, − 0.70 [ − 0.81, − 0.58], *p* < 0.0001; met-DR, − 0.50 [−  0.70, − 0.29], *p* < 0.0001) (Fig. 3).

#### Mean Body Weight and BMI

**Fig. 4:**
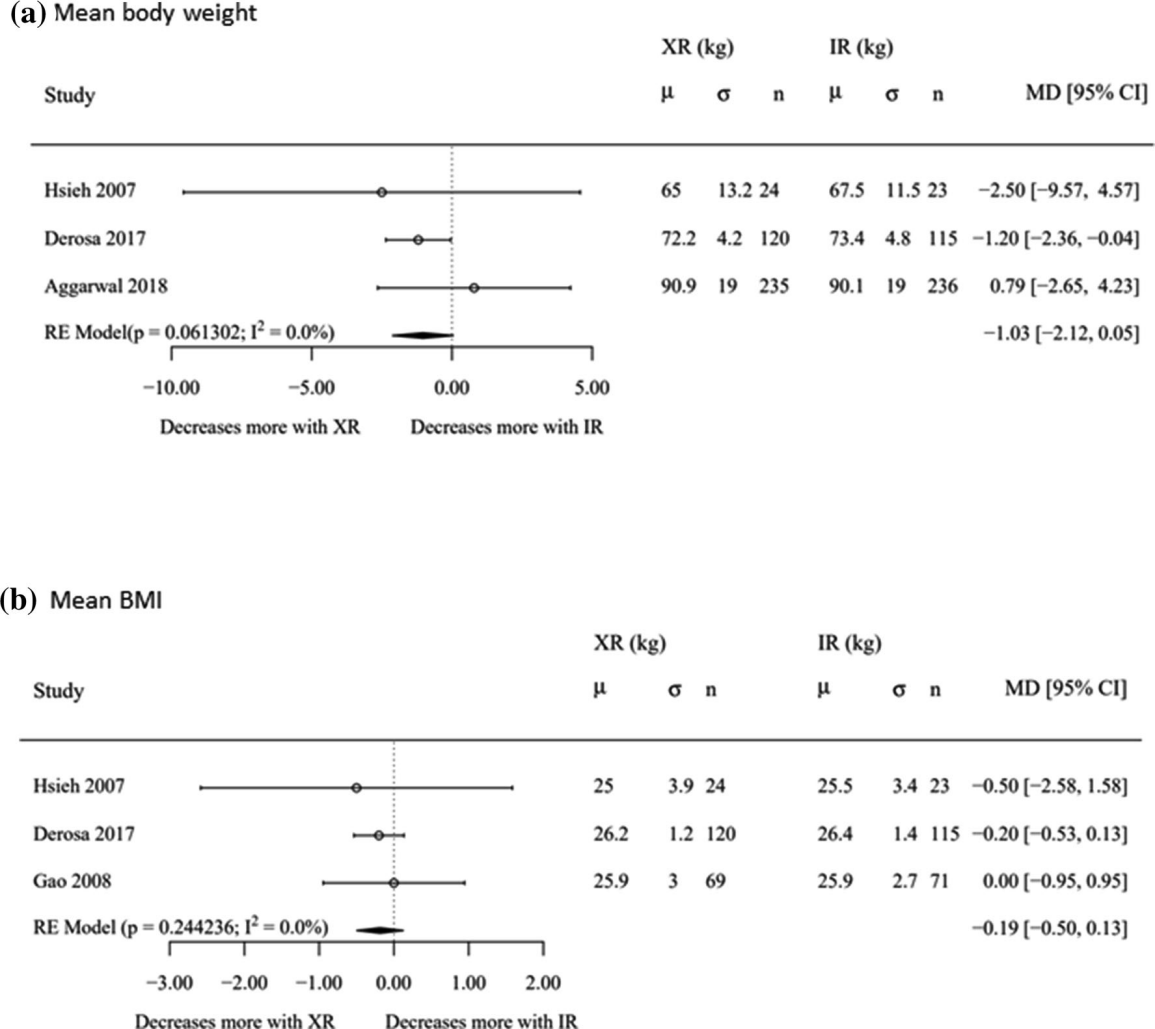
Effect of metformin formulations upon **a** mean body weight (kg) and **b** mean BMI (kg/m^2^). Only comparisons available were met-XR versus met-IR. Mean difference and 95% confidence intervals

Three studies including 829 participants reported mean body weight for met-IR vs. met-XR. There was a non-significant decrease in post-treatment mean body weight in individuals randomised to met-XR vs. met-IR (− 1.03 [− 2.12, 0.05], *p* = 0.06) (Fig. 4a). Three studies (*n* = 430) reported post-treatment BMI for met-IR vs. met-XR. There was no significant difference in post-treatment BMI between participants randomised to met-XR v. met-IR (Fig. 4b). No included studies involving met-DR reported these outcomes.

#### Lipid Profile

There were no significant differences in the concentrations of total cholesterol, HDL cholesterol or triglycerides observed in participants randomised to met-XR versus met-IR (Table 1). Individuals randomised to met-XR had significantly decreased post-treatment LDL cholesterol concentrations compared to those randomised to met-IR (Table 1).

**Table 1:** Post-treatment lipid profile

Lipid parameter	Mean diff	[95% CI]	Study no. (*n*)	*I*^2^ (%)	*P* value
Total cholesterol	– 2.90	[– 6.09, 0.30]	6 (1831)	44	0.08
HDL cholesterol	0.25	[– 0.61, 1.11]	4 (1560)	0	0.56
LDL cholesterol	– 5.50	[– 8.08, – 2.92]	4 (1550)	30	< 0.0001
Triglycerides	1.22	[– 3.28, 5.72)	6 (1725)	29	0.60

Lipid levels (mg/dl)

All comparisons are met-DR v. met-IR. No studies comparing met-DR v. met-IR or met-DR vs. met-XR reported post-treatment lipid profiles

*diff* difference, *met* metformin, *DR* delayed-release, *XR* extended-release, *IR* immediate-release

### Side Effects of Metformin Formulations

**Fig. 5:**
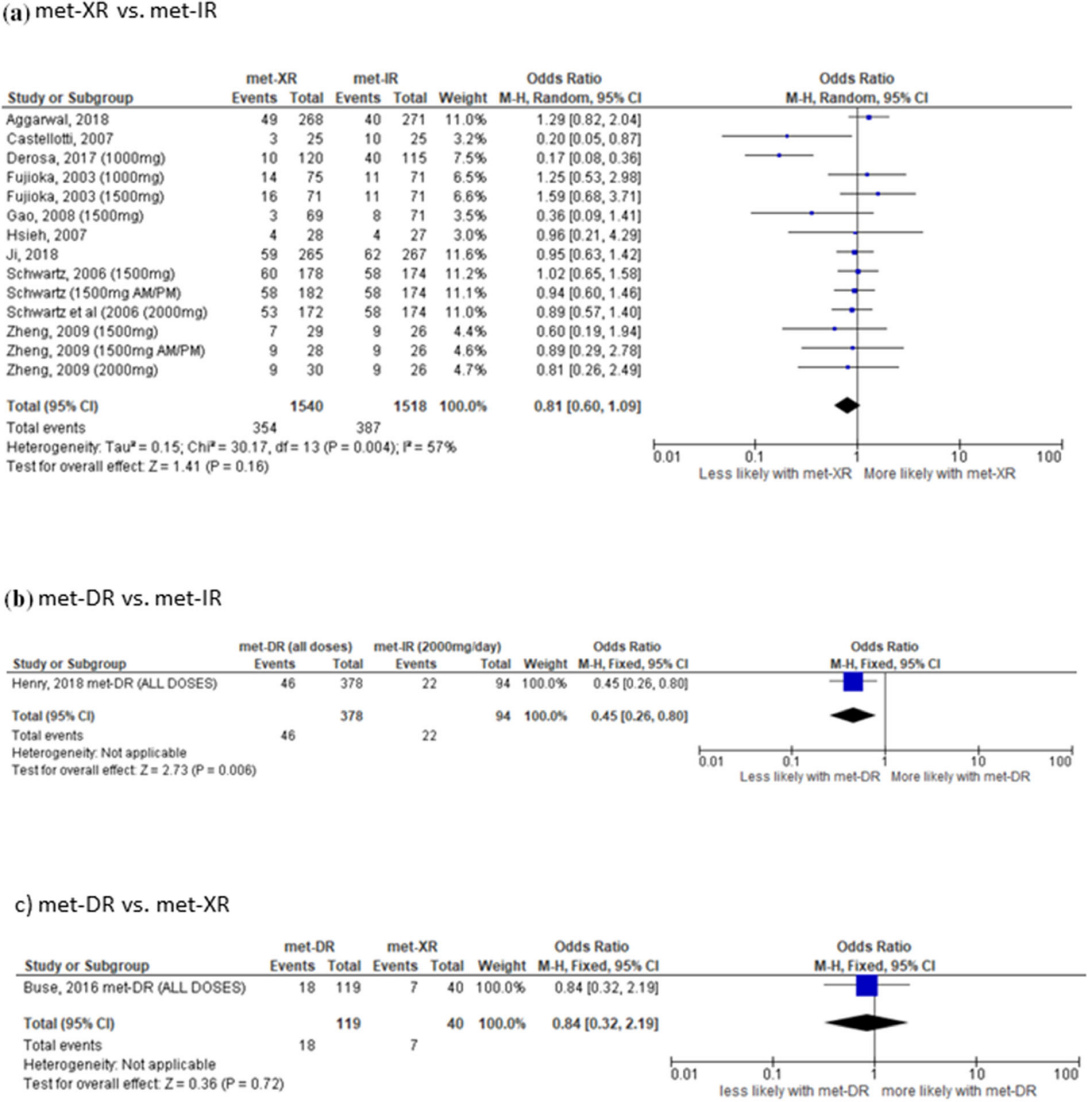
Effect of metformin formulations upon overall gastrointestinal side effects in **a** met-XR versus met-IR, **b** met-DR vs. met-IR and **c** met-DR versus met-XR. Odds ratio and 95% confidence interval

There was a non-statistically significant reduction in the likelihood of experiencing GI side effects between individuals randomised to met-XR versus met-IR (OR 0.69, 95% CI 0.45–1.07, *I*^2^ = 71%, *p* = 0.10), based on nine studies including 2164 participants (Fig. 5a). Individuals randomised to met-DR versus met-IR were less than half as likely to experience any GI side effects (OR 0.45, 95% CI 0.26–0.80, *I*^2^ = N/A, *p* = 0.006), based on one study including 472 participants (Fig. 5b). There was no significant difference in the likelihood of developing any GI side effects between individuals randomised to met-DR versus met-XR (OR 0.84, 95% CI 0.32–2.19, *I*^2^ = N/A, *p* = 0.72), in one study including 159 participants (Fig. 5c). When each reported side effect was considered separately, randomisation to met-XR versus met-IR was associated with reduced likelihood of heartburn/dyspepsia and increased tolerability overall (Table 2). Randomisation to met-DR v met-IR was associated with reduced likelihood of nausea (Table 2). No studies comparing met-DR versus met-XR reported the likelihood of experiencing each side effect separately.

**Table 2:** Separate side effects

Adverse effect	Comparison	Mean diff [95% CI]	Study no. (patients)	*P* value	*I*^2^ (%)
Diarrhoea	met-XR vs. met-IR	0.87 [0.43, 1.41]	8 (2063)	0.41	64
	met-DR vs. met-IR	0.67 [0.34, 1.31]	1 (477)	0.24	N/A
Vomiting	met-XR vs. met-IR	0.90 [0.37, 2.18]	3 (920)	0.82	30
	met-DR vs. met-IR		–	–	–
Nausea	met-XR vs. met-IR	0.83 [0.57, 1.21]	7 (2036)	0.34	0
	met-DR vs. met-IR	0.25 [0.12, 0.52]	1 (475)	0.004	0
Abdominal pain/bloating	met-XR vs. met-IR	1.24 [0.76, 2.03]	7 (2011)	0.39	0
	met-DR vs. met-IR	–	–	–	–
Flatulence	met-XR vs. met-IR	0.71 [0.09, 5.44]	3 (526)	0.75	66
	met-DR vs. met-IR	–	–	–	–
Heartburn/dyspepsia	met-XR vs. met-IR	0.49 [0.25, 0.97]	4	0.04	0
	met-DR vs. met-IR	–	–	–	–
Headache	met-XR vs. met-IR	0.99 [0.39, 2.52]	2	0.98	0
	met-DR vs. met-IR	–	–	–	–
Overall drug tolerance	met-XR vs. met-IR	0.25 [0.16, 0.38]	1	< 0.00001	N/A
	met-DR vs. met-IR	–	–	–	–

*diff* difference, *met* metformin, *DR* delayed-release, *XR* extended-release, *IR* immediate-release

## Discussion

### Main Findings

Our findings suggest that in people with T2DM and/or PCOS there were no significant differences in efficacy of glycaemic control between all included metformin formulations (IR, XR and DR), based on change in FPG and in HbA1c after treatment. In addition, our analyses showed very similar reductions in FPG with randomisation to met-XR versus met-DR across a range of clinically relevant doses. There was no significant difference in the magnitude of weight loss experienced by patients randomised to different metformin formulations or in the change in their BMI during treatment. However, patients randomised to met-XR had significantly reduced LDL cholesterol levels compared to those randomised to met-IR. GI side effects were markedly reduced in patients randomised to longer-acting metformin preparations compared to met-IR, particularly with met-DR.

### Strengths

Our analyses provides the first systematic comparison of the three formulations of metformin that have currently been trialled in head-to-head trials. Meta-regression and extensive sensitivity analyses have been performed to optimise comparisons between available studies and to leverage all existing data, despite methodological differences between the original studies, for example in dosing and study length. There are few previous syntheses of data directly comparing preparations of metformin. A previous comprehensive review concluded that side effects were reduced with met-XR compared to IR, but predated the availability of the new met-DR formulation [35].

### Limitations

The drawing of definitive conclusions from our meta-analysis was limited by both the quantity and quality of the studies available. In particular, there was a lack of studies involving comparisons of individuals randomised to met-DR vs. met-IR (one study; *n* = 571) [20] or met-DR vs. met-XR (two studies; *n* = 260) [19, 34]. We thus suggest a cautious view regarding the interpretation of these comparisons. Our results highlight the need for more high-quality studies investigating optimal use of various metformin formulations, particularly in patient populations other than type 2 diabetes. Moreover, if more and higher quality data were available then non-significant effects, for example reduction in total cholesterol levels with met-XR v. met-IR, might reach significance, as would be expected from the observed reduction in LDL cholesterol. While metformin is prescribed for a range of clinical indications, the studies available for inclusion in our meta-analysis were limited mainly to populations with T2DM. We were thus only able to compare the efficacy of different metformin formulations for outcomes related to T2DM: glycaemic control, body weight and lipid profiles.

### Interpretation

Despite the frequent and widespread prescription of metformin worldwide, there is a relative lack of head-to-head comparison data regarding optimal formulations. This information is important to help guide decision-making about the cost–benefit ratio of prescribing long-acting metformin preparations; in some circumstances the relatively high upfront cost of these preparations may make their use prohibitive, despite potential clinical advantages [36]. The equal efficacies of the different metformin formulations in lowering glucose, despite significant differences in drug plasma concentrations, may be explained at least in part by the importance of the gut-based mechanisms of action of metformin, for example stimulating glucagon-like peptide 1 (GLP-1)/peptide YY secretion, alterations of bile acid metabolism and potentially changes to the gut microbiome [17].

In our analysis, mean body weight was reduced following treatment with metformin, regardless of formulation. The underlying mechanism of metformin-associated weight loss remains poorly understood and is likely multifactorial, with several possible contributing mechanisms. Metformin-associated weight loss may be centrally mediated via suppression of orexigenic hypothalamic neurons potentially via GDF-15 [37], by decreased expression of neuropeptide Y, or by preventing ghrelin-mediated appetite stimulation [38]. There is likely also to be an important contribution from gut-based mechanisms, including increased secretion of GLP-1 [39]. It has also been suggested that GI side effects may contribute to metformin-associated weight loss via malabsorption of bile salts, microbiome alteration or and gut serotonin secretion [39]. Although we found that long-acting preparations of metformin were associated with reduced GI side effects compared to immediate-release metformin, there was no significant difference in the magnitude of weight loss associated with long-acting preparations, suggesting that GI side effects were not the main mediators of weight loss.

Metformin improves lipid profiles by decreasing the activity and expression of several products involved in lipid synthesis, including acetyl CoA carboxylase (ACCase), sterol regulatory element-binding protein 1 (SREBP-1), fatty acid synthase (FAS) and HMG CoA reductase (HMGCR) [39]. We found that people randomised to met-XR versus met-IR had reduced LDL cholesterol levels, suggesting that long-acting metformin may show some benefit over the immediate-release formulation in improving long-term cardio-metabolic risk profiles.

## Conclusions

Our results demonstrate equal efficacy of longer-acting (XR, DR) versus IR metformin formulations in terms of glycaemic control, but significant additional advantages with the longer-acting formulations. Metformin XR was associated with reduced serum LDL cholesterol concentrations, while metformin DR was strongly associated with reduced GI side effects, which could improve drug compliance. Further research is required to refine the optimal cost–benefit ratio of the different available preparations of metformin in various clinical circumstances.

## Supplementary Information

Below is the link to the electronic supplementary material.

Supplementary file1 (XLSX 51 kb)

Supplementary file2 (PDF 3077 kb)
