# Supplementary material for: Efficacy and Side Effect Profile of Different Formulations of Metformin: A Systematic Review and Meta-Analysis
Source: Diabetes Ther. 2021 Jun 2;12(7):1901–14. doi: 10.1007/s13300-021-01058-2 (PMC8266931; doi:10.1007/s13300-021-01058-2)
Supplement: Supplementary file 2 — Supplementary file2 (PDF 3077 kb) [file 13300_2021_1058_MOESM2_ESM.pdf]

**Supplementary S1 Fig: PRISMA flow diagram**

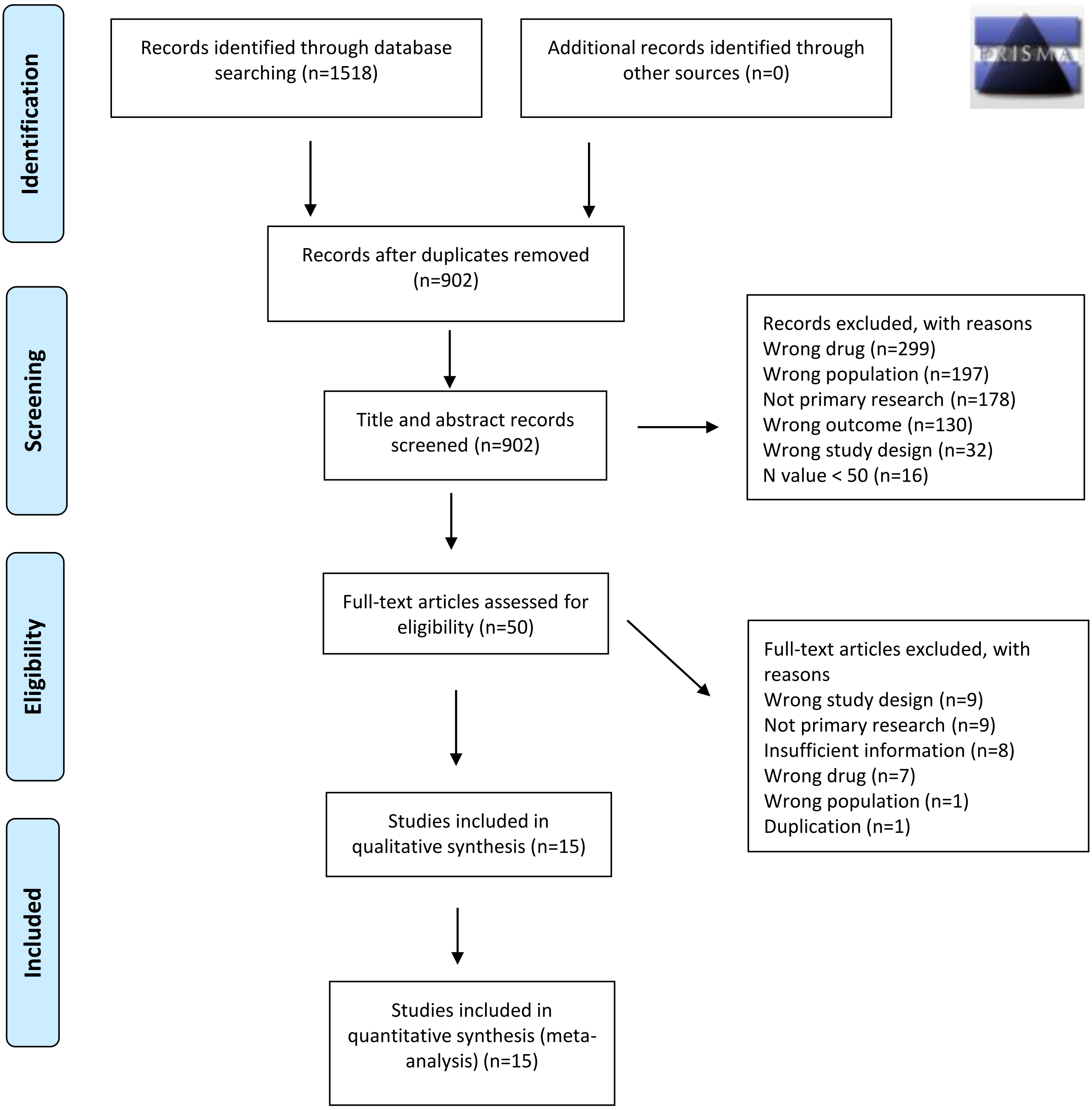

**Supplementary Fig S2: LOO sensitivity analysis**

A) Mean body weight

| Study or Subgroup | Mean Difference | SE | Weight | Mean Difference IV, Fixed, 95% CI |
|---|---|---|---|---|
| All studies | -1.23 | 0.571429 | 29.8% | -1.23 [-2.35, -0.11] |
| minus Aggarwal, 2018 | -1.01 | 0.540816 | 33.2% | -1.01 [-2.07, 0.05] |
| minus Derosa, 2017 | 0.23 | 1.492347 | 4.4% | 0.23 [-2.69, 3.15] |
| minus Hsieh, 2007 | -0.98 | 0.545918 | 32.6% | -0.98 [-2.05, 0.09] |
| **Total (95% CI)** | | | **100.0%** | **-1.01 [-1.62, -0.40]** |

Heterogeneity: $Chi^2 = 0.84$, df = 3 (P = 0.84); $I^2 = 0\%$
Test for overall effect: Z = 3.24 (P = 0.001)

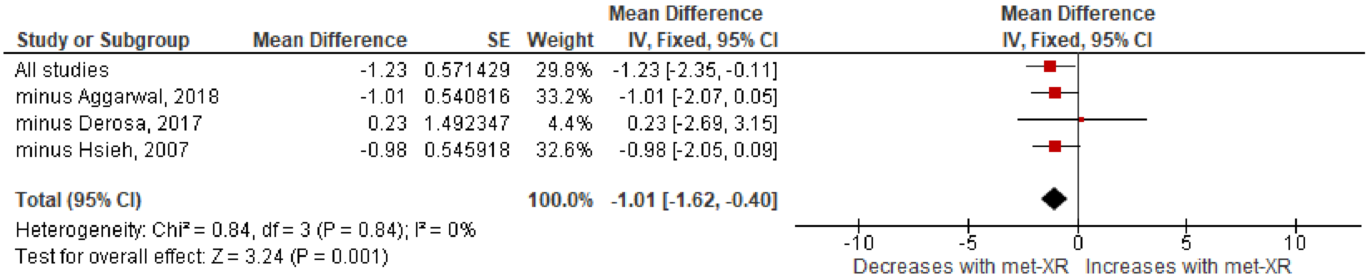


B) Mean BMI

| Study or Subgroup | Mean Difference | SE | Weight | Mean Difference IV, Fixed, 95% CI |
|---|---|---|---|---|
| All studies | -0.18 | 0.173469 | 33.4% | -0.18 [-0.52, 0.16] |
| minus Derosa, 2017 | -0.09 | 0.441327 | 5.2% | -0.09 [-0.95, 0.77] |
| minus Gao, 2008 | -0.21 | 0.186224 | 29.0% | -0.21 [-0.57, 0.15] |
| minus Hsieh, 2007 | -0.17 | 0.17602 | 32.4% | -0.17 [-0.51, 0.17] |
| **Total (95% CI)** | | | **100.0%** | **-0.18 [-0.38, 0.02]** |

Heterogeneity: $Chi^2 = 0.07$, df = 3 (P = 1.00); $I^2 = 0\%$
Test for overall effect: Z = 1.80 (P = 0.07)

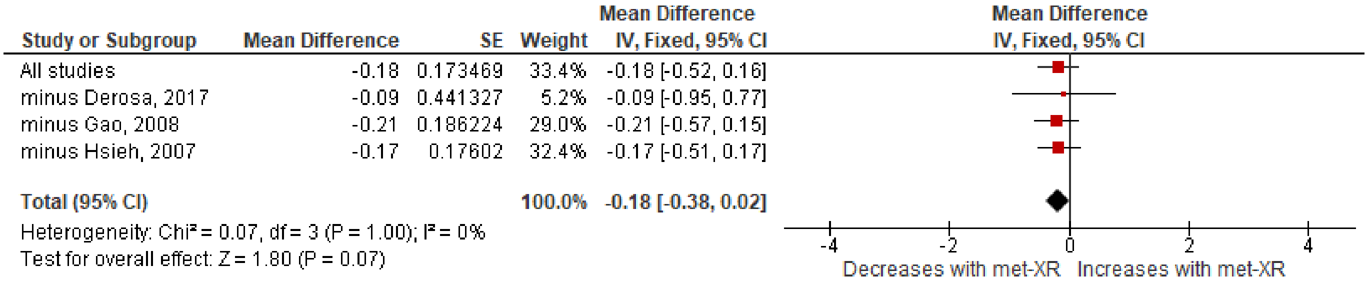


C) Total GI adverse events

| Study or Subgroup | log[Odds Ratio] | SE | Weight | Odds Ratio IV, Fixed, 95% CI |
|---|---|---|---|---|
| All studies | -0.21467016 | 0.163265 | 10.8% | 0.81 [0.59, 1.11] |
| minus Aggarwal, 2018 | -0.16115091 | 0.158163 | 11.5% | 0.85 [0.62, 1.16] |
| minus Castellotti, 2007 | -0.11918641 | 0.173347 | 9.6% | 0.89 [0.63, 1.25] |
| minus Derosa, 2017 (1000mg) | -0.02687215 | 0.158163 | 11.5% | 0.97 [0.71, 1.33] |
| minus Fujioka, 2003 (1500mg) | -0.19382003 | 0.160714 | 11.1% | 0.82 [0.60, 1.13] |
| minus Gao, 2008 (1500mg) | -0.13667714 | 0.173469 | 9.6% | 0.87 [0.62, 1.23] |
| minus Hsieh, 2007 (2000mg) | -0.16749109 | 0.165816 | 10.5% | 0.85 [0.61, 1.17] |
| minus Ji, 2018 | -0.19382003 | 0.188776 | 8.1% | 0.82 [0.57, 1.19] |
| minus Schwartz, 2006 met-XR (ALL DOSES) | -0.19382003 | 0.191327 | 7.9% | 0.82 [0.57, 1.20] |
| minus Zheng, 2009 (ALL DOSES) | -0.16749109 | 0.173469 | 9.6% | 0.85 [0.60, 1.19] |
| **Total (95% CI)** | | | **100.0%** | **0.86 [0.77, 0.95]** |

Heterogeneity: $Chi^2 = 1.00$, df = 9 (P = 1.00); $I^2 = 0\%$
Test for overall effect: Z = 2.89 (P = 0.004)

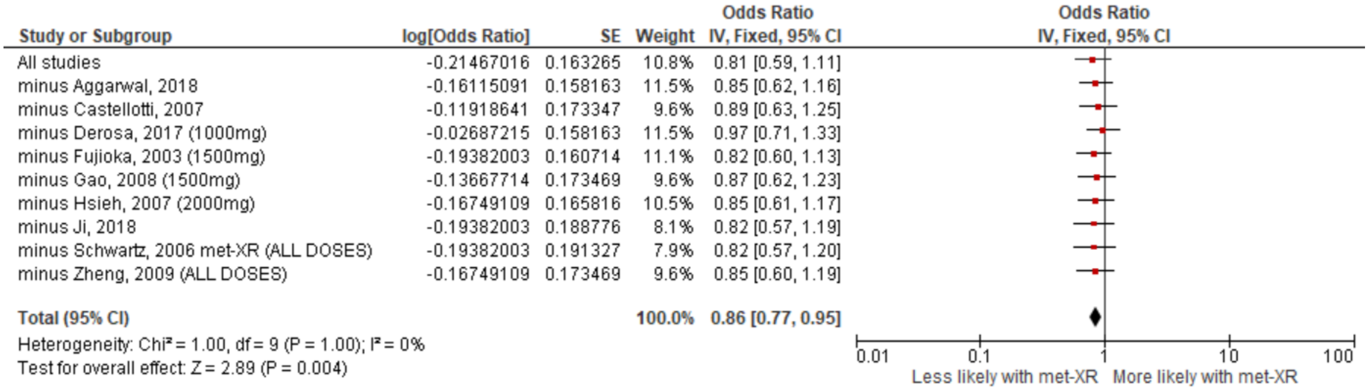

D) Diarrhoea

| Study or Subgroup | log[Odds Ratio] | SE | Weight | Odds Ratio IV, Fixed, 95% CI |
|---|---|---|---|---|
| All studies | -0.1079054 | 0.25 | 12.2% | 0.90 [0.55, 1.47] |
| minus Aggarwal, 2018 | -0.1739252 | 0.283163 | 9.5% | 0.84 [0.48, 1.46] |
| minus Derosa, 2017 (1000mg) | -0.16749109 | 0.262755 | 11.1% | 0.85 [0.51, 1.42] |
| minus Fujioka, 2003 (1500mg) | -0.16749109 | 0.193878 | 20.3% | 0.85 [0.58, 1.24] |
| minus Gao, 2008 (1500mg) | -0.07572071 | 0.265306 | 10.9% | 0.93 [0.55, 1.56] |
| minus Hameed, 2017 (2000mg) | -0.01772877 | 0.27551 | 10.1% | 0.98 [0.57, 1.69] |
| minus Ji, 2018 | -0.13667714 | 0.321429 | 7.4% | 0.87 [0.46, 1.64] |
| minus Schwartz, 2006 met-XR (ALL DOSES) | -0.18045606 | 0.295918 | 8.7% | 0.83 [0.47, 1.49] |
| minus Zheng, 2009 (ALL DOSES) | -0.09691001 | 0.280612 | 9.7% | 0.91 [0.52, 1.57] |
| **Total (95% CI)** | | | **100.0%** | **0.88 [0.74, 1.04]** |

Heterogeneity: Chi² = 0.34, df = 8 (P = 1.00); I² = 0%
Test for overall effect: Z = 1.46 (P = 0.14)

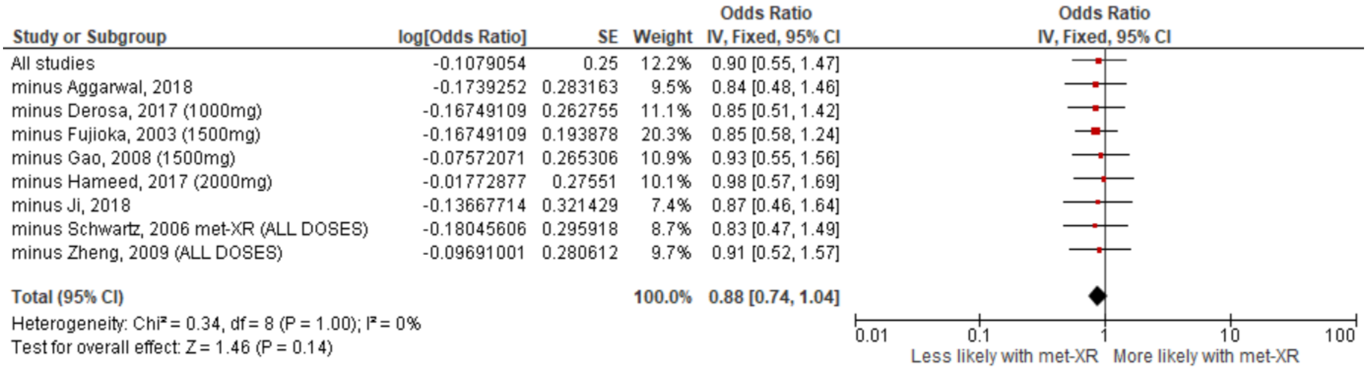


E) Vomiting

| Study or Subgroup | log[Odds Ratio] | SE | Weight | Odds Ratio IV, Fixed, 95% CI |
|---|---|---|---|---|
| All studies | -0.04575749 | 0.461735 | 30.1% | 0.96 [0.39, 2.36] |
| minus Aggarwal, 2018 | -0.43179828 | 0.390306 | 42.2% | 0.65 [0.30, 1.40] |
| minus Derosa, 2017 (1000mg) | 0.11394335 | 0.780612 | 10.5% | 1.12 [0.24, 5.18] |
| minus Fujioka, 2003 (1500mg) | 0 | 0.612245 | 17.1% | 1.00 [0.30, 3.32] |
| **Total (95% CI)** | | | **100.0%** | **0.83 [0.51, 1.37]** |

Heterogeneity: Chi² = 0.73, df = 3 (P = 0.87); I² = 0%
Test for overall effect: Z = 0.73 (P = 0.47)

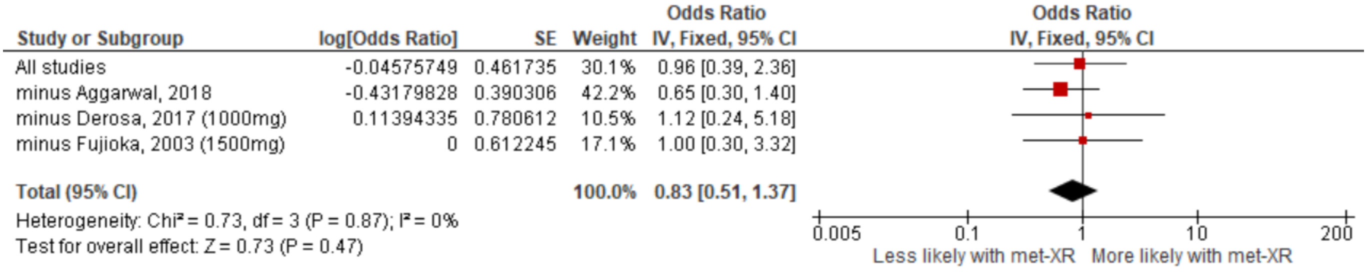


F) Nausea

| Study or Subgroup | log[Odds Ratio] | SE | Weight | Odds Ratio IV, Fixed, 95% CI |
|---|---|---|---|---|
| All studies | -0.08092191 | 0.163265 | 11.7% | 0.92 [0.67, 1.27] |
| minus Aggarwal, 2018 | -0.15490196 | 0.155612 | 12.9% | 0.86 [0.63, 1.16] |
| minus Derosa, 2017 (1000mg) | -0.08092191 | 0.165816 | 11.3% | 0.92 [0.67, 1.28] |
| minus Fujioka, 2003 (1500mg) | -0.07572071 | 0.170918 | 10.7% | 0.93 [0.66, 1.30] |
| minus Gao, 2008 (1500mg) | -0.07572071 | 0.168367 | 11.0% | 0.93 [0.67, 1.29] |
| minus Ji, 2018 | -0.06048075 | 0.196429 | 8.1% | 0.94 [0.64, 1.38] |
| minus Schwartz, 2006 met-XR (ALL DOSES) | -0.05060999 | 0.229592 | 5.9% | 0.95 [0.61, 1.49] |
| minus Zheng, 2009 (ALL DOSES) | -0.06550155 | 0.104592 | 28.5% | 0.94 [0.76, 1.15] |
| **Total (95% CI)** | | | **100.0%** | **0.92 [0.83, 1.03]** |

Heterogeneity: Chi² = 0.28, df = 7 (P = 1.00); I² = 0%
Test for overall effect: Z = 1.46 (P = 0.14)

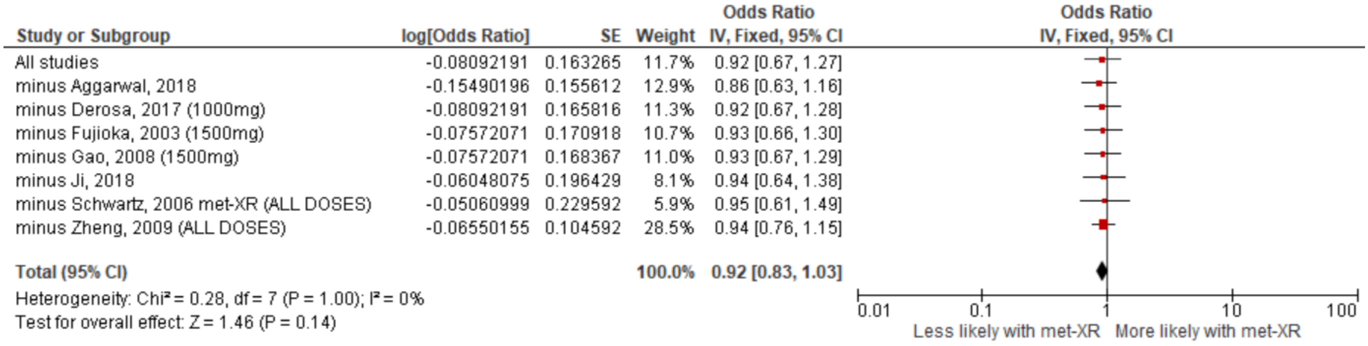

G) Abdominal pain/bloating

| Study or Subgroup | log[Odds Ratio] | SE | Weight | Odds Ratio IV, Fixed, 95% CI |
|---|---|---|---|---|
| minus Aggarwal, 2018 | 0.09342169 | 0.32398 | 16.3% | 1.10 [0.58, 2.07] |
| minus Derosa, 2017 (1000mg) | 0.07918125 | 0.339286 | 14.8% | 1.08 [0.56, 2.10] |
| minus Fujioka, 2003 (1500mg) | 0.09691001 | 0.331633 | 15.5% | 1.10 [0.58, 2.11] |
| minus Gao, 2008 (1500mg) | 0.08635983 | 0.318878 | 16.8% | 1.09 [0.58, 2.04] |
| minus Ji, 2018 | 0.10037055 | 0.336735 | 15.1% | 1.11 [0.57, 2.14] |
| minus Schwartz, 2006 met-XR (ALL DOSES) | 0.17897695 | 0.558673 | 5.5% | 1.20 [0.40, 3.57] |
| minus Zheng, 2009 (ALL DOSES) | 0.04532298 | 0.326531 | 16.0% | 1.05 [0.55, 1.98] |
| **Total (95% CI)** | | | **100.0%** | **1.09 [0.85, 1.41]** |

Heterogeneity: Chi² = 0.05, df = 6 (P = 1.00); I² = 0%
Test for overall effect: Z = 0.68 (P = 0.50)

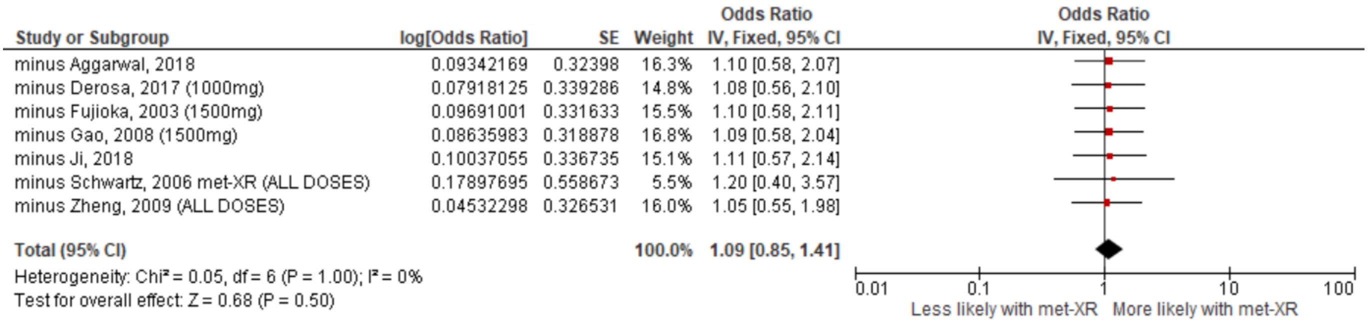


H) Heartburn/dyspepsia

| Study or Subgroup | log[Odds Ratio] | SE | Weight | Odds Ratio IV, Fixed, 95% CI |
|---|---|---|---|---|
| All studies | -0.30980392 | 0.183673 | 25.9% | 0.73 [0.51, 1.05] |
| minus Fujioka, 2003 (1500mg) | -0.30103 | 0.206633 | 20.5% | 0.74 [0.49, 1.11] |
| minus Hameed, 2017 (2000mg) | -0.24412514 | 0.27551 | 11.5% | 0.78 [0.46, 1.34] |
| minus Schwartz, 2006 met-XR (ALL DOSES) | -0.36653154 | 0.227041 | 16.9% | 0.69 [0.44, 1.08] |
| minus Zheng, 2009 (ALL DOSES) | -0.32790214 | 0.186224 | 25.2% | 0.72 [0.50, 1.04] |
| **Total (95% CI)** | | | **100.0%** | **0.73 [0.61, 0.88]** |

Heterogeneity: Chi² = 0.13, df = 4 (P = 1.00); I² = 0%
Test for overall effect: Z = 3.37 (P = 0.0008)

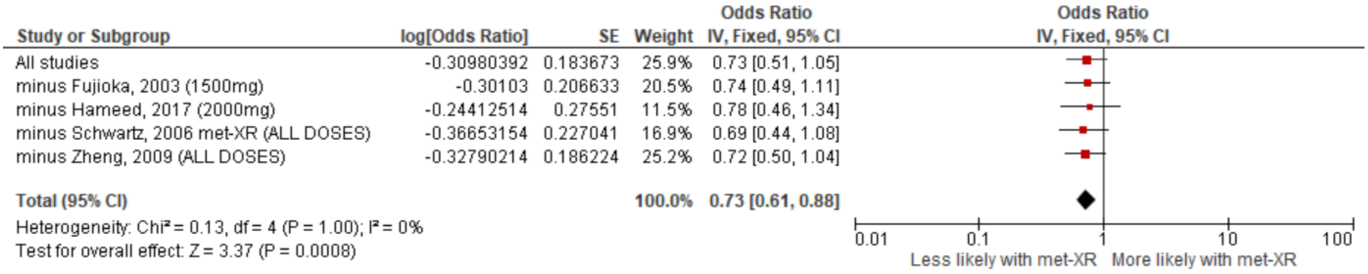


I) Flatulence

| Study or Subgroup | log[Odds Ratio] | SE | Weight | Odds Ratio IV, Fixed, 95% CI |
|---|---|---|---|---|
| All studies | -0.35654732 | 1.262755 | 30.9% | 0.70 [0.06, 8.32] |
| minus Derosa, 2017 (1000mg) | 0.18184359 | 2.34949 | 8.9% | 1.20 [0.01, 119.91] |
| minus Fujioka, 2003 (1500mg) | -0.67778071 | 0.951531 | 54.4% | 0.51 [0.08, 3.28] |
| minus Gao, 2008 (1500mg) | -0.49485002 | 2.920918 | 5.8% | 0.61 [0.00, 186.80] |
| **Total (95% CI)** | | | **100.0%** | **0.61 [0.15, 2.42]** |

Heterogeneity: Chi² = 0.13, df = 3 (P = 0.99); I² = 0%
Test for overall effect: Z = 0.70 (P = 0.48)

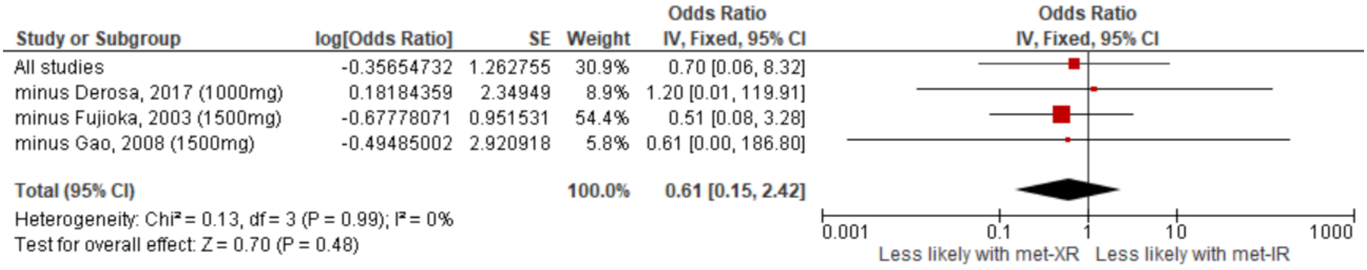


J) Headache

| Study or Subgroup | log[Odds Ratio] | SE | Weight | Odds Ratio IV, Fixed, 95% CI |
|---|---|---|---|---|
| All studies | -0.0043648 | 0.543367 | 56.5% | 1.00 [0.34, 2.89] |
| minus Aggarwal, 2018 | -0.02687215 | 1.188776 | 11.8% | 0.97 [0.09, 10.01] |
| minus Fujioka, 2003 | 0.00432137 | 0.72449 | 31.8% | 1.00 [0.24, 4.15] |
| **Total (95% CI)** | | | **100.0%** | **1.00 [0.45, 2.22]** |

Heterogeneity: Chi² = 0.00, df = 2 (P = 1.00); I² = 0%
Test for overall effect: Z = 0.01 (P = 0.99)

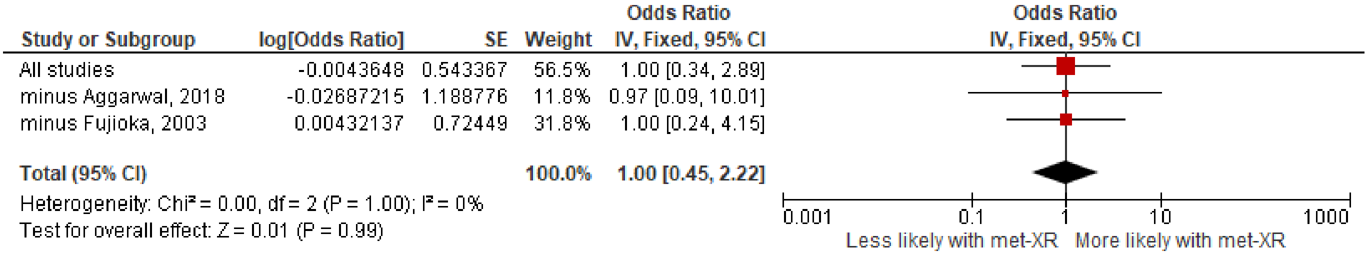

K) Total cholesterol (weeks 12 & 24)

| Study or Subgroup | Mean Difference | SE | Weight | Mean Difference IV, Fixed, 95% CI |
|---|---|---|---|---|
| All studies | -2.9 | 1.630102 | 16.7% | -2.90 [-6.09, 0.29] |
| minus Aggarwal, 2018 | -3.62 | 1.821429 | 13.4% | -3.62 [-7.19, -0.05] |
| minus Derosa, 2017 (1000mg) | -0.17 | 2.007653 | 11.0% | -0.17 [-4.10, 3.76] |
| minus Gao, 2008 (1500mg) | -3.57 | 1.681122 | 15.7% | -3.57 [-6.86, -0.28] |
| minus Hsieh, 2007 (2000mg) | -3.2 | 1.645408 | 16.4% | -3.20 [-6.42, 0.02] |
| minus Schwartz, 2006 met-XR (ALL DOSES) | -3.17 | 1.688776 | 15.5% | -3.17 [-6.48, 0.14] |
| minus Zheng, 2009 (ALL DOSES) | -2.94 | 1.971939 | 11.4% | -2.94 [-6.80, 0.92] |
| **Total (95% CI)** | | | **100.0%** | **-2.90 [-4.20, -1.59]** |

Heterogeneity: Chi² = 2.22, df = 6 (P = 0.90); I² = 0%
Test for overall effect: Z = 4.35 (P < 0.0001)

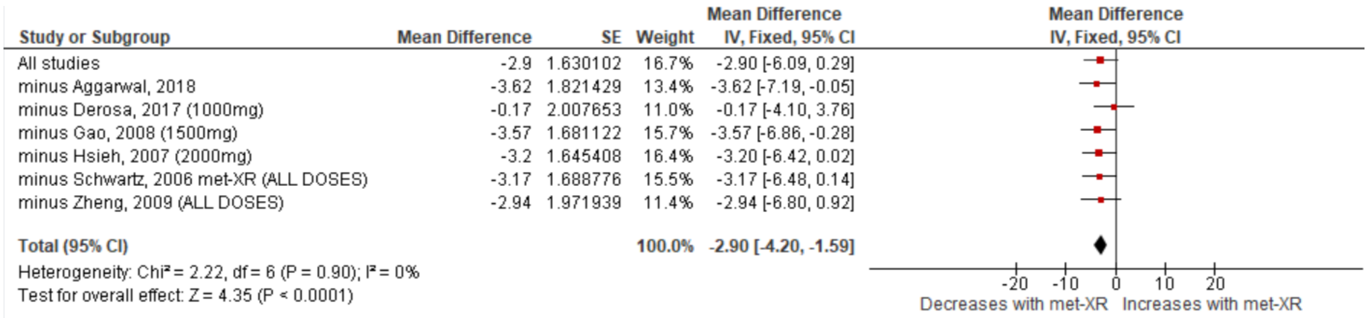


L) HDL cholesterol (weeks 12 & 24)

| Study or Subgroup | Mean Difference | SE | Weight | Mean Difference IV, Fixed, 95% CI |
|---|---|---|---|---|
| All studies | 0.13 | 0.563776 | 25.0% | 0.13 [-0.97, 1.23] |
| minus Aggarwal, 2018 | 0.41 | 0.645408 | 19.1% | 0.41 [-0.85, 1.67] |
| minus Derosa, 2017 (1000mg) | -0.04 | 0.716837 | 15.5% | -0.04 [-1.44, 1.36] |
| minus Gao, 2008 (1500mg) | 0.13 | 0.581633 | 23.5% | 0.13 [-1.01, 1.27] |
| minus Schwartz, 2006 met-XR (ALL DOSES) | -0.05 | 0.686224 | 16.9% | -0.05 [-1.39, 1.29] |
| **Total (95% CI)** | | | **100.0%** | **0.13 [-0.43, 0.68]** |

Heterogeneity: Chi² = 0.31, df = 4 (P = 0.99); I² = 0%
Test for overall effect: Z = 0.45 (P = 0.65)

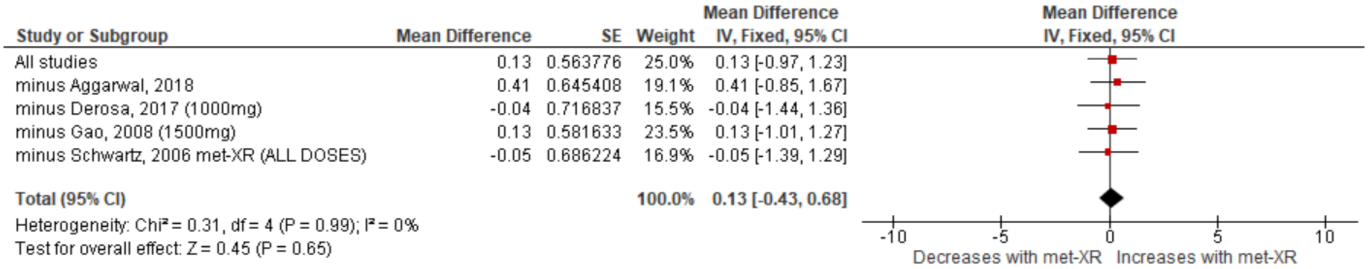


M) LDL cholesterol (weeks 12 & 24)

| Study or Subgroup | Mean Difference | SE | Weight | Mean Difference IV, Fixed, 95% CI |
|---|---|---|---|---|
| All studies | -5.5 | 1.316327 | 25.1% | -5.50 [-8.08, -2.92] |
| minus Aggarwal, 2018 | -6.35 | 1.420918 | 21.5% | -6.35 [-9.13, -3.57] |
| minus Derosa, 2017 (1000mg) | -2.43 | 2.25551 | 8.5% | -2.43 [-6.85, 1.99] |
| minus Gao, 2008 (1500mg) | -5.86 | 1.359694 | 23.5% | -5.86 [-8.52, -3.20] |
| minus Schwartz, 2006 met-XR (ALL DOSES) | -5.47 | 1.423469 | 21.4% | -5.47 [-8.26, -2.68] |
| **Total (95% CI)** | | | **100.0%** | **-5.50 [-6.79, -4.21]** |

Heterogeneity: Chi² = 2.28, df = 4 (P = 0.68); I² = 0%
Test for overall effect: Z = 8.35 (P < 0.00001)

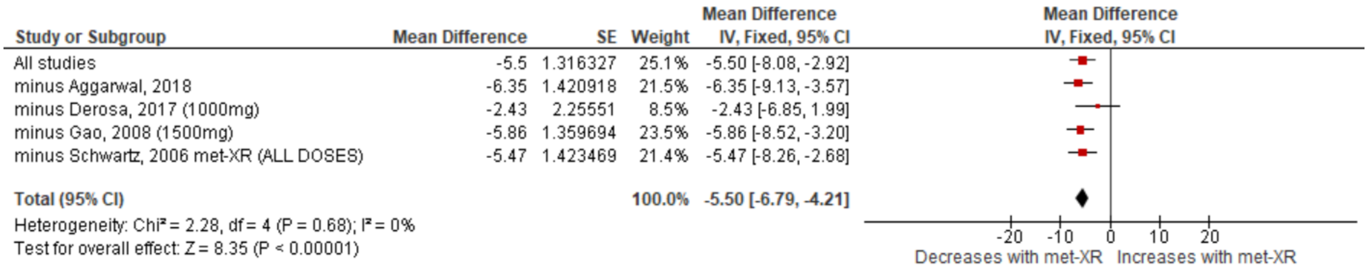

N) Triglycerides (weeks 12 & 24)

| Study or Subgroup | Mean Difference | SE | Weight | Mean Difference IV, Random, 95% CI |
|---|---|---|---|---|
| minus Aggarwal, 2018 | 1.22 | 2.295918 | 18.2% | 1.22 [-3.28, 5.72] |
| minus Derosa, 2017 (1000mg) | 0.6 | 2.372449 | 17.1% | 0.60 [-4.05, 5.25] |
| minus Gao, 2008 (1500mg) | 3.62 | 2.563776 | 14.6% | 3.62 [-1.40, 8.64] |
| minus Hsieh, 2007 (2000mg) | 0.87 | 2.357143 | 17.3% | 0.87 [-3.75, 5.49] |
| minus Schwartz, 2006 met-XR (ALL DOSES) | 1.3 | 2.298469 | 18.2% | 1.30 [-3.20, 5.80] |
| minus Zheng, 2009 (ALL DOSES) | -0.96 | 2.568878 | 14.6% | -0.96 [-5.99, 4.07] |
| **Total (95% CI)** | | | **100.0%** | **1.10 [-0.82, 3.02]** |

Heterogeneity: Tau² = 0.00; Chi² = 1.67, df = 5 (P = 0.89); I² = 0%
Test for overall effect: Z = 1.12 (P = 0.26)

Mean Difference IV, Random, 95% CI

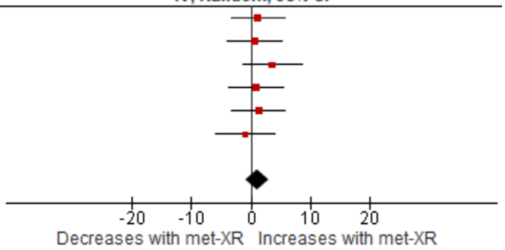


O) Mean FPG (start & end points) (weeks 12 & 24)

(i) met-XR

| Study or Subgroup | Mean Difference | SE | Weight | Mean Difference IV, Fixed, 95% CI |
|---|---|---|---|---|
| All studies | -21.9 | 5.127551 | 14.7% | -21.90 [-31.95, -11.85] |
| Buse, 2016 met-DR (ALL DOSES) | -23.7268 | 5.778195 | 11.6% | -23.73 [-35.05, -12.40] |
| minus Derosa, 2017 (1000mg) | -21.6939 | 6.020386 | 10.7% | -21.69 [-33.49, -9.89] |
| minus Gao, 2008 (1500mg) | -25.2441 | 3.888591 | 25.5% | -25.24 [-32.87, -17.62] |
| minus Hameed, 2017 (2000mg) | -22.235 | 5.757914 | 11.6% | -22.23 [-33.52, -10.95] |
| minus Schwartz, 2006 met-XR (ALL DOSES) | -20.941 | 5.552293 | 12.5% | -20.94 [-31.82, -10.06] |
| minus Zheng, 2009 (ALL DOSES) | -19.7746 | 5.371718 | 13.4% | -19.77 [-30.30, -9.25] |
| **Total (95% CI)** | | | **100.0%** | **-22.58 [-26.43, -18.73]** |

Heterogeneity: Chi² = 0.91, df = 6 (P = 0.99); I² = 0%
Test for overall effect: Z = 11.49 (P < 0.00001)

Mean Difference IV, Fixed, 95% CI

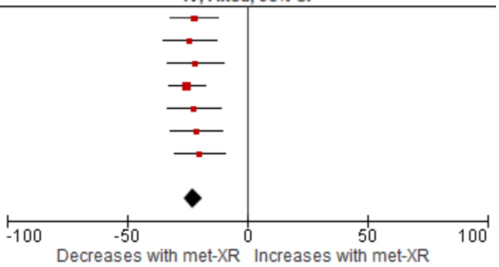


(ii) met-IR

| Study or Subgroup | Mean Difference | SE | Weight | Mean Difference IV, Fixed, 95% CI |
|---|---|---|---|---|
| All studies | -23.2 | 12.3699 | 14.5% | -23.20 [-47.44, 1.04] |
| MInus Gao, 2008 (1500mg) | -34.4388 | 5.736025 | 67.2% | -34.44 [-45.68, -23.20] |
| Minus Schwartz, 2006 (1500mg) | -18.3904 | 15.24307 | 9.5% | -18.39 [-48.27, 11.49] |
| Minus Zheng, 2009 (1500mg) | -17.5496 | 15.86808 | 8.8% | -17.55 [-48.65, 13.55] |
| **Total (95% CI)** | | | **100.0%** | **-29.80 [-39.02, -20.58]** |

Heterogeneity: Chi² = 2.09, df = 3 (P = 0.55); I² = 0%
Test for overall effect: Z = 6.34 (P < 0.00001)

Mean Difference IV, Fixed, 95% CI

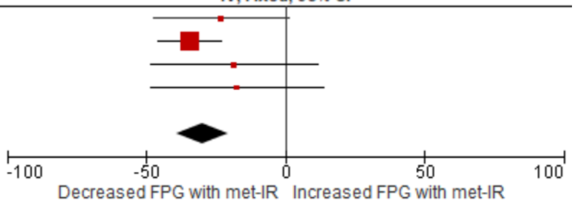

(iii) met-DR

| Study or Subgroup | Mean Difference | SE | Weight | Mean Difference IV, Fixed, 95% CI |
|---|---|---|---|---|
| All studies | -20 | 2.882653 | 27.7% | -20.00 [-25.65, -14.35] |
| Buse, 2016 met-DR (ALL DOSES) | -18.8675 | 2.515966 | 36.4% | -18.87 [-23.80, -13.94] |
| Henry, 2018 met-DR (ALL DOSES) | -18.7545 | 2.535826 | 35.8% | -18.75 [-23.72, -13.78] |
| **Total (95% CI)** | | | **100.0%** | **-19.14 [-22.12, -16.17]** |

Heterogeneity: Chi² = 0.12, df = 2 (P = 0.94); I² = 0%
Test for overall effect: Z = 12.61 (P < 0.00001)

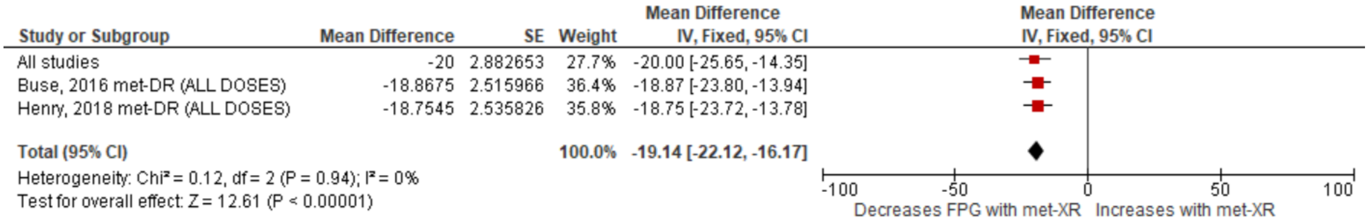


(P) Mean Hb1Ac (start & end points)

(i) met-XR

| Study or Subgroup | Mean Difference | SE | Weight | Mean Difference IV, Fixed, 95% CI |
|---|---|---|---|---|
| All studies | -0.66 | 0.058 | 10.0% | -0.66 [-0.77, -0.55] |
| minus Buse, 2016 met-DR (ALL DOSES) | -0.64045 | 0.08485 | 4.7% | -0.64 [-0.81, -0.47] |
| minus Derosa, 2017 (1000mg) | -0.76117 | 0.031833 | 33.1% | -0.76 [-0.82, -0.70] |
| minus Gao, 2008 (1500mg) | -0.74915 | 0.03142 | 33.9% | -0.75 [-0.81, -0.69] |
| minus Hameed, 2017 (2000mg) | -0.67114 | 0.071793 | 6.5% | -0.67 [-0.81, -0.53] |
| minus Schwartz, 2006 met-XR (ALL DOSES) | -0.69653 | 0.065026 | 7.9% | -0.70 [-0.82, -0.57] |
| minus Zheng, 2009 (ALL DOSES) | -0.645 | 0.09159 | 4.0% | -0.65 [-0.82, -0.47] |
| **Total (95% CI)** | | | **100.0%** | **-0.73 [-0.76, -0.69]** |

Heterogeneity: Chi² = 5.65, df = 6 (P = 0.46); I² = 0%
Test for overall effect: Z = 39.66 (P < 0.00001)

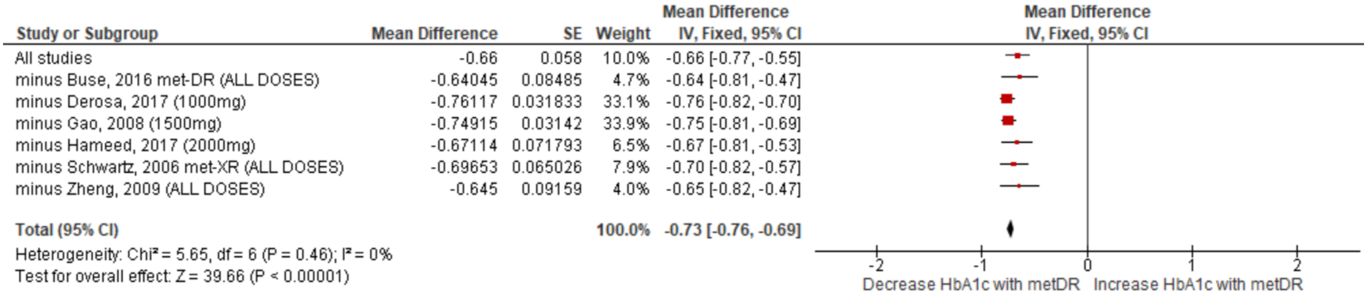


(ii) met-IR

| Study or Subgroup | Mean Difference | SE | Weight | Mean Difference IV, Fixed, 95% CI |
|---|---|---|---|---|
| All studies | -0.7 | 0.196429 | 16.6% | -0.70 [-1.08, -0.32] |
| MInus Gao, 2008 (1500mg) | -0.77576 | 0.099033 | 65.4% | -0.78 [-0.97, -0.58] |
| Minus Schwartz, 2006 (1500mg) | -0.53983 | 0.219105 | 13.4% | -0.54 [-0.97, -0.11] |
| Minus Zheng, 2009 (1500mg) | -0.63735 | 0.373105 | 4.6% | -0.64 [-1.37, 0.09] |
| **Total (95% CI)** | | | **100.0%** | **-0.73 [-0.88, -0.57]** |

Heterogeneity: Chi² = 1.05, df = 3 (P = 0.79); I² = 0%
Test for overall effect: Z = 9.06 (P < 0.00001)

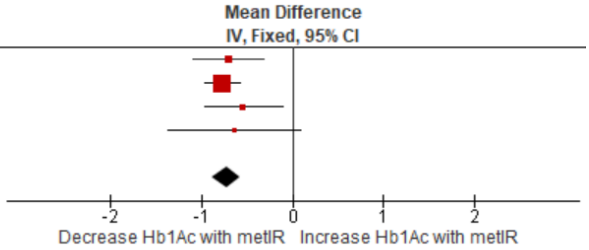


(iii) met-DR

| Study or Subgroup | Mean Difference | SE | Weight | Mean Difference IV, Fixed, 95% CI |
|---|---|---|---|---|
| All studies | -0.5 | 0.104592 | 41.0% | -0.50 [-0.70, -0.30] |
| Buse, 2016 met-DR (ALL DOSES) | -0.50551 | 0.109648 | 37.3% | -0.51 [-0.72, -0.29] |
| Henry, 2018 met-DR (ALL DOSES) | -0.50146 | 0.143949 | 21.7% | -0.50 [-0.78, -0.22] |
| **Total (95% CI)** | | | **100.0%** | **-0.50 [-0.63, -0.37]** |

Heterogeneity: Chi² = 0.00, df = 2 (P = 1.00); I² = 0%
Test for overall effect: Z = 7.50 (P < 0.00001)

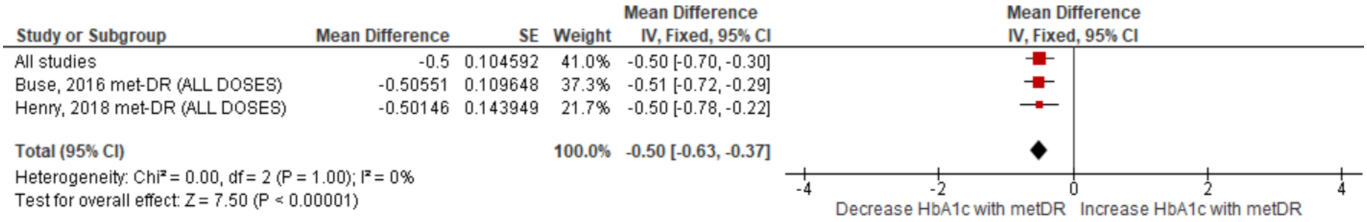

**Supplementary Fig. S3: Funnel Plots**

A) Fasting plasma glucose (met-XR vs. met-IR)

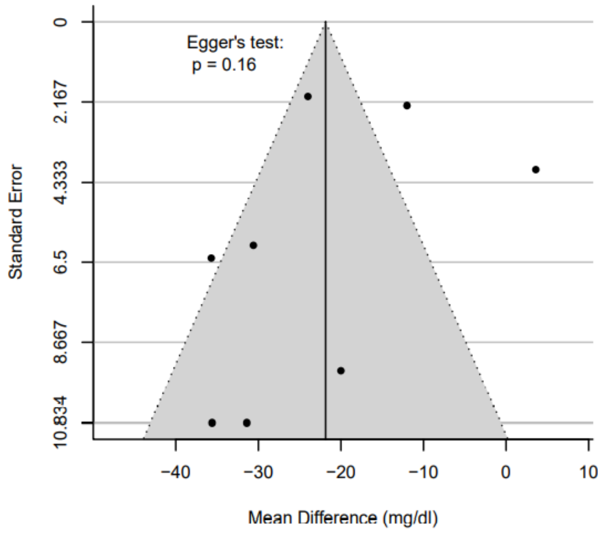


B) Fasting plasma glucose (met-DR vs. met-IR)

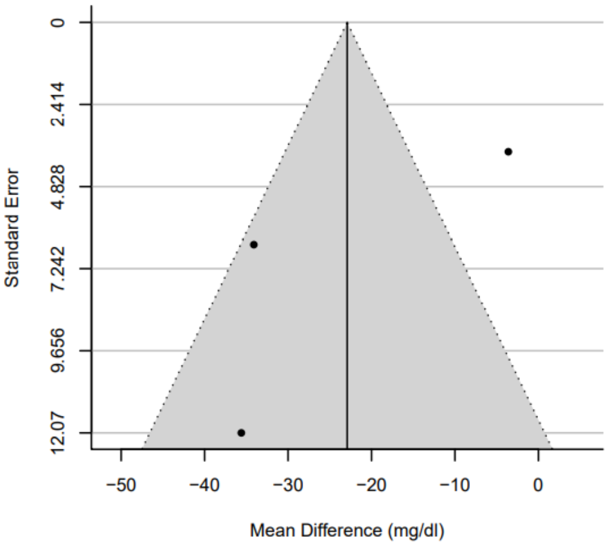


C) Fasting plasma glucose (met-DR vs. met-XR)

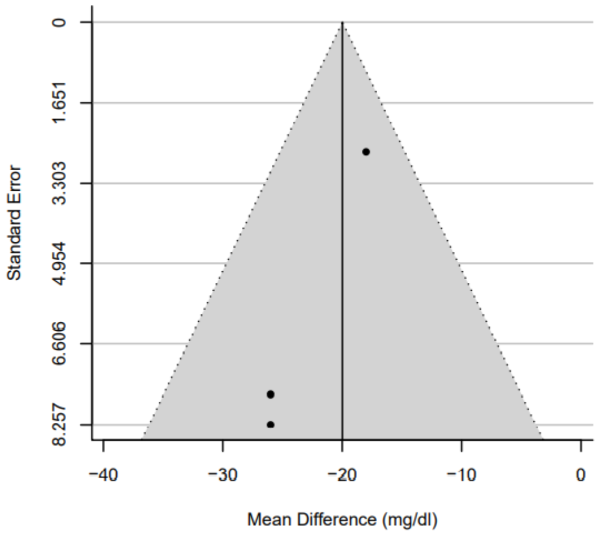


D) HbA1c (met-XR vs. met-IR)

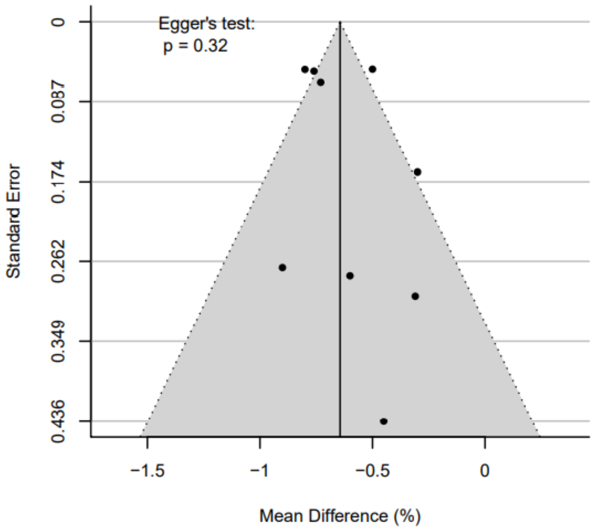

E) HbA1c (met-DR vs. met-IR)

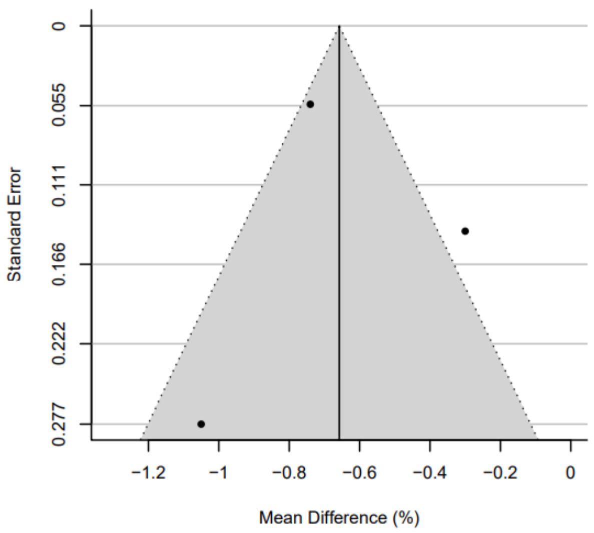


F) HbA1c (met-DR vs. met-XR)

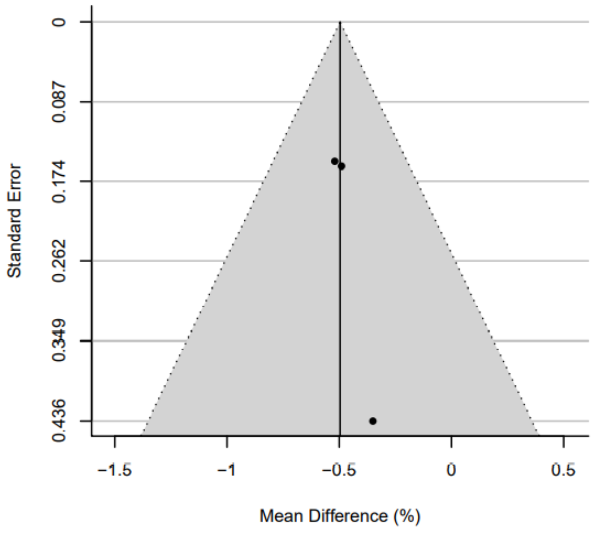


G) Mean body weight (kg)

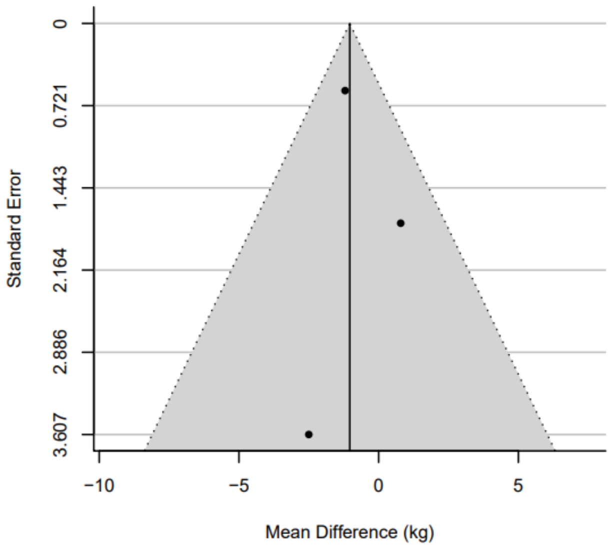


H) Mean BMI (kg/m2)

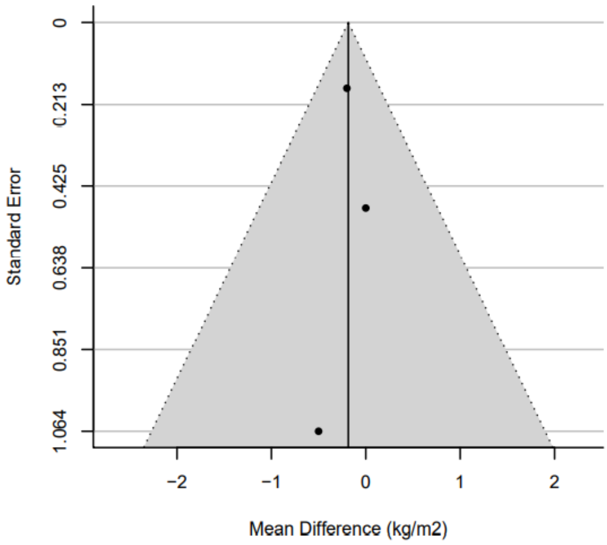

I) All gastro-intestinal adverse effects (met-XR vs. met-IR)

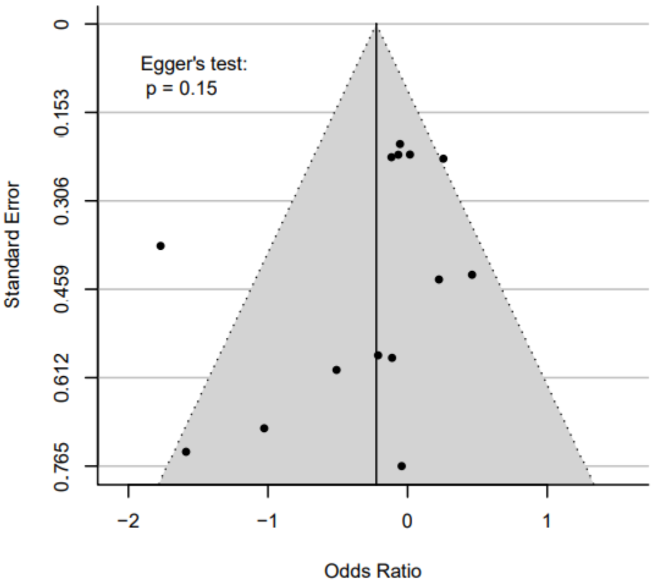


J) All gastrointestinal adverse effects (met-DR vs. met-IR)

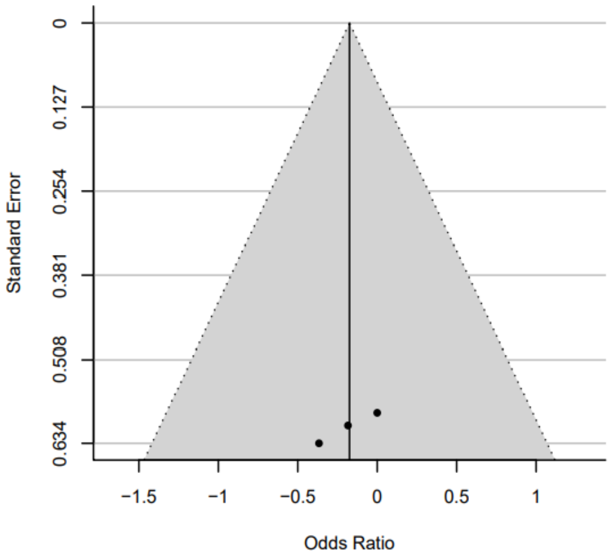


K) Total cholesterol (met-XR vs. met-IR)

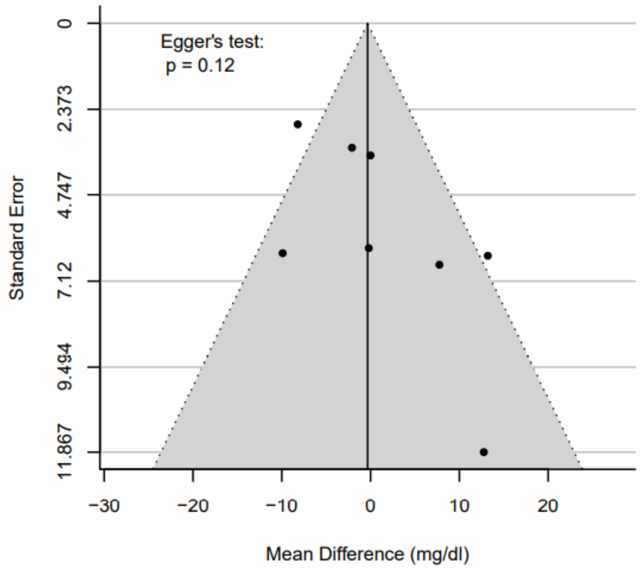


L) HDL cholesterol (met-XR vs. met-IR)

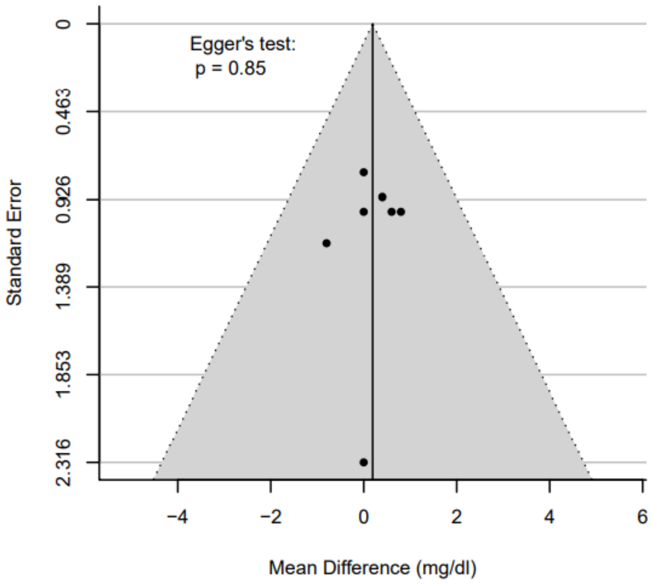

M) HDL cholesterol (met-XR vs. met-IR)

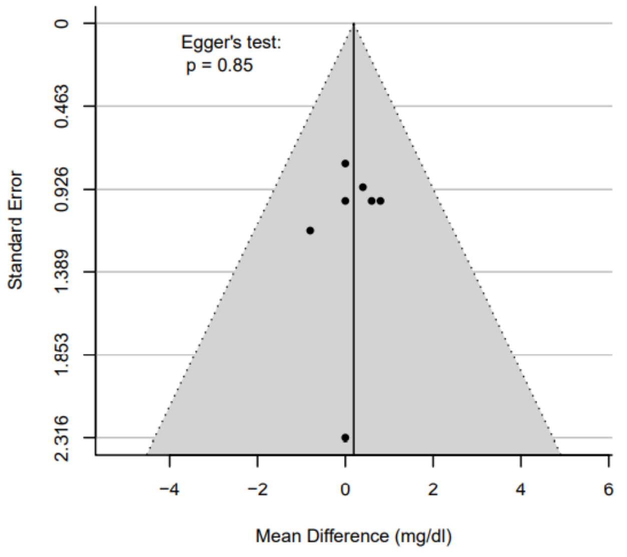


N) LDL cholesterol (met-XR vs. met-IR)

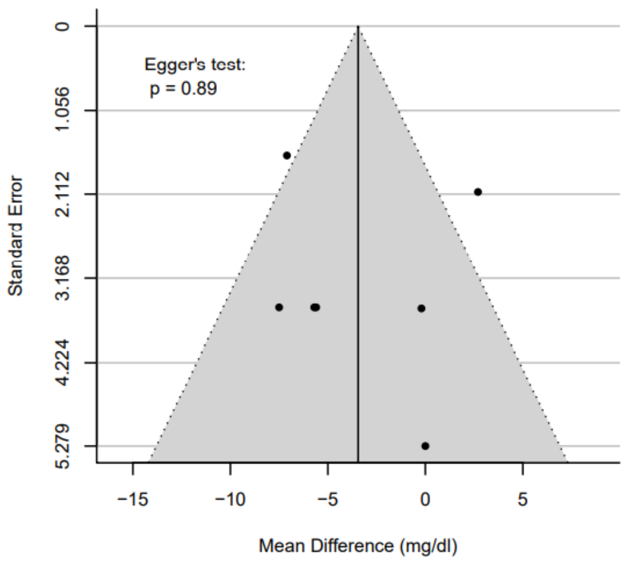


O) Triglycerides (met-XR vs. met-IR)

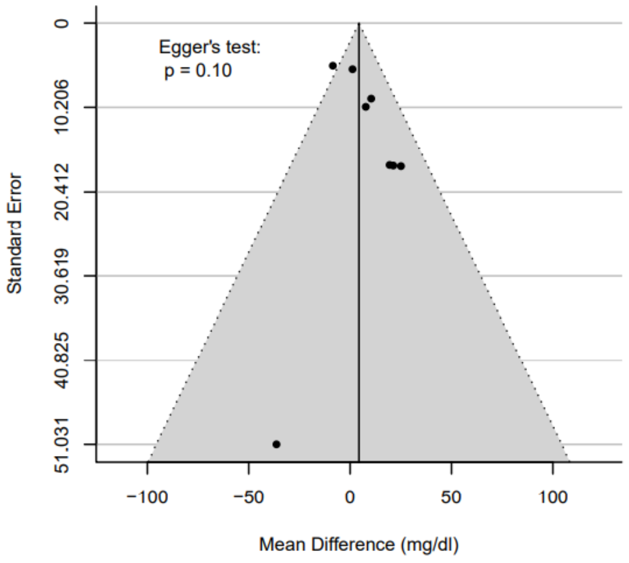

# Supplementary Fig S4: GRADE Analysis

| Outcomes | Plain language statements | Absolute Effect | | | Relative effect (95% CI) | Certainty of the evidence GRADE |
|---|---|---|---|---|---|---|
| | | With immediate-effect metformin | With sustained metformin formulation | | | |
| FBS<br>Follow-up: 0 | *(1) Risk of Bias: Studies in this outcome were deemed to have a low risk of bias, with only 1 out of 10 studies (33 groups) not-intention-to-treat. (Removal of this study did not alter this outcome). (2) Imprecision: 3 studies out of 10 in this outcome had large 95% confidence intervals, therefore reducing the precision of this outcome. (3) Inconsistency: Studies in this outcome were deemed to have a very high level of heterogeneity as evidenced by the I2 value ranging from 9.7% to 90.6%, (dependent upon comparison), with accompanying p value of <0.00001. (4) Indirectness: Participants and interventions in all studies in this outcome did not differ from those of interest. This outcome was also specified in the PROSPERO protocol. (5) Publication bias: All studies for this outcome were judged to have a low publication bias, due no obvious asymmetries in Funnels plots for any study outcomes and Eggers Testing.* | 135.3 mg/dl | 132.65 mg/dl | Average difference (MD): 2.65 mg/dl lower<br>(95% CI: 6.32 mg/dl lower to 1.03 mg/dl higher)<br>Based on data from 1470 patients in 9 studies | - | ⊕⊕⊕◯<br>MODERATE<br>Due to serious inconsistency. |
| HBA1c | *(1) Risk of Bias: Studies in this outcome were deemed to have a low risk of bias, with 1 out of 9 studies not-intention-to-treat. Removal of these studies did not alter the outcome. (2) Imprecision: 2 studies out of 9 in this outcome had large 95% confidence intervals, therefore reducing the precision of this outcome. (3) Inconsistency: met-IR and met-XR studies were deemed to have a high level of heterogeneity, whereas and met-DR demonstrated a low level of heterogeneity as evidenced by I2 values of 83%, 51.3% and 0% respectively. ( (4) Indirectness: Participants and interventions in all studies in this outcome did not differ from those of interest. This outcome was also specified in the PROSPERO protocol. (5) Publication bias: All studies for this outcome were judged to have a low publication bias, due no obvious asymmetries in Funnels plots for any study outcomes and Eggers Testing.* | 7 mg/dl | 6.89 mg/dl | Average difference (MD): 0.11 mg/dl lower<br>(95% CI: 0.2 to 0.02 mg/dl lower)<br>Based on data from 1144 patients in 9 studies | - | ⊕⊕⊕◯<br>MODERATE<br>Due to serious inconsistency. |
| Mean body weight<br>Follow-up: 0 | *(1) Risk of Bias: Studies in this outcome were deemed to have a low risk of bias as all studies were intention to treat. (2) Imprecision: This outcome was judged to have a high level of imprecision due to the low number of studies (3) and participants (374). The majority (2) also had large 95% confidence intervals. (3) Inconsistency: Studies in this outcome were deemed to have low level of heterogeneity as evidenced by the I2 value of 0%). (4) Indirectness: Participants and interventions in all studies in this outcome did not differ from those of interest. This outcome was also specified in the PROSPERO protocol. (5) Publication bias: All studies for this outcome were judged to have a low publication bias, due no obvious asymmetries in Funnels plots for any study outcomes and Eggers Testing.* | 77 kg | 75.99 kg | Average difference (MD): 1.01 kg lower<br>(95% CI: 2.07 kg lower to 0.05 kg higher)<br>Based on data from 829 patients in 3 studies | - | ⊕⊕⊕◯<br>MODERATE<br>Due to serious imprecision. |
| Mean BMI<br>Follow-up: 0 | *(1) Risk of Bias: Studies in this outcome were deemed to have a low risk of bias with none of the 3 studies not intention-to-treat. (2) Imprecision: This outcome was judged to have a high level of imprecision due to the low number of studies (3) and participants (209). Two out of these studies had large 95% confidence intervals. (3) Inconsistency: Studies in this outcome were deemed to have a low level of heterogeneity as evidenced by the I2 value of 0%. (4) Indirectness: Participants and interventions in all studies in this outcome did not differ from those of interest. This outcome was also specified in the PROSPERO protocol. (5) Publication bias: All studies for this outcome were judged to have a low publication bias, due no obvious asymmetries in Funnels plots for any study outcomes and Eggers Testing.* | 25.9 kg/m2 | 25.71 kg/m2 | Average difference (MD): 0.19 kg/m2 lower<br>(95% CI: 0.49 kg/m2 lower to 0.12 kg/m2 higher)<br>Based on data from 430 patients in 3 studies | - | ⊕⊕⊕◯<br>MODERATE<br>Due to serious imprecision. |
| Total cholesterol<br>Follow-up: 0 | *(1) Risk of Bias: Studies in this outcome were deemed to have a low risk of bias with none of the 6 studies not intention-to-treat. (2) Imprecision: This outcome was judged to have a low level of imprecision due to the acceptable number of studies (6) and participants (2225). One of these studies had a large 95% confidence interval. (3) Inconsistency: Studies in this outcome were deemed to have a moderate level of heterogeneity as evidenced by the I2 value of 53% and p value of 0.02. (4) Indirectness: Participants and interventions in all studies in this outcome did not differ from those of interest. This outcome was also specified in the PROSPERO protocol. (5) Publication bias: All studies for this outcome were judged to have a low publication bias, due no obvious asymmetries in Funnels plots for any study outcomes and Eggers Testing.* | 201.7 mg/dl | 199.98 mg/dl | Average difference (MD): 1.72 mg/dl lower<br>(95% CI: 5.63 mg/dl lower to 2.19 mg/dl higher)<br>Based on data from 2225 patients in 10 studies | - | |
| HDL cholesterol<br>Follow-up: 0 | *(1) Risk of Bias: Studies in this outcome were deemed to have a low risk of bias with none of the 6 studies not intention-to-treat. (2) Imprecision: This outcome was judged to have a low level of imprecision due to the acceptable number of studies (6) and participants (2225). One of these studies had a large 95% confidence interval. (3) Inconsistency: Studies in this outcome were deemed to have a moderate level of heterogeneity as evidenced by the I2 value of 53% and p value of 0.02. (4) Indirectness: Participants and interventions in all studies in this outcome did not differ from those of interest. This outcome was also specified in the PROSPERO protocol. (5) Publication bias: All studies for this outcome were judged to have a low publication bias, due no obvious asymmetries in Funnels plots for any study outcomes and Eggers Testing.* | 47.6 mg/dl | 47.85 mg/dl | Average difference (MD): 0.25 mg/dl higher<br>(95% CI: 0.61 mg/dl lower to 1.11 mg/dl higher)<br>Based on data from 1902 patients in 6 studies | - | ⊕⊕⊕◯<br>MODERATE<br>Due to serious inconsistency. |
| LDL cholesterol<br>Follow-up: 0 | *(1) Risk of Bias: Studies in this outcome were deemed to have a low risk of bias with none of the 6 studies not intention-to-treat. (2) Imprecision: This outcome was judged to have a low level of imprecision due to the acceptable number of studies (6) and participants (1884). All studies were considered to have modest 95% confidence intervals. (3) Inconsistency: Studies in this outcome were deemed to have a low level of heterogeneity as evidenced by the I2 value of 0% and p value of 0.47. (4) Indirectness: Participants and interventions in all studies in this outcome did not differ from those of interest. This outcome was also specified in the PROSPERO protocol. (5) Publication bias: All studies for this outcome were judged to have a low publication bias, due no obvious asymmetries in Funnels plots for any study outcomes and Eggers Testing.* | 114 mg/dl | 108.27 mg/dl | Average difference (MD): 5.73 mg/dl lower<br>(95% CI: 7.91 to 3.56 mg/dl lower)<br>Based on data from 1884 patients in 6 studies | - | ⊕⊕⊕⊕<br>HIGH |
| Triglycerides<br>Follow-up: 0 | *(1) Risk of Bias: Studies in this outcome were deemed to have a low risk of bias with none of the 6 studies not intention-to-treat. (2) Imprecision: This outcome was judged to have a low level of imprecision due to the acceptable number of studies (5) and participants (1303). All studies, but one were considered to have small 95% confidence intervals. (3) Inconsistency: Studies in this outcome were deemed to have a low level of heterogeneity as evidenced by the I2 value of 23% and p value of 0.23. (4) Indirectness: Participants and interventions in all studies in this outcome did not differ from those of interest. This outcome was also specified in the PROSPERO protocol. (5) Publication bias: All studies for this outcome were judged to have a low publication bias, due no obvious asymmetries in Funnels plots for any study outcomes and Eggers Testing.* | 123.5 mg/dl | 125.81 mg/dl | Average difference (MD): 2.31 mg/dl higher<br>(95% CI: 1.68 mg/dl lower to 6.29 mg/dl higher)<br>Based on data from 2115 patients in 10 studies | - | ⊕⊕⊕⊕<br>HIGH |
| Total GI side effects | *(1) Risk of Bias: Studies in this outcome were deemed to have a low risk of bias with 2 studies out of 9 not intention-to-treat (for the met-XR vs. met-IR comparison). Removal of these studies did not alter this outcome. Studies in other comparisons were all intention to treat. (2) Imprecision: This outcome was judged to have an overall high level of imprecision due to the moderate number of studies (9) and participants (2101) in the met-XR vs. met-IR comparison, yet only 1 study in the met-DR vs. met-IR and the met-DR vs. met-XR comparisons. The 3 of these studies had large 95% confidence intervals. (3) Inconsistency: Studies in this outcome were deemed to have a relatively high level of heterogeneity as evidenced by the I2 value of 57% for the met-XR vs. met-IR comparison, with accompanying p value of 0.004. (4) Indirectness: Participants and interventions in all studies in this outcome did not differ from those of interest. This outcome was also specified in the PROSPERO protocol. (5) Publication bias: All studies for this outcome were judged to have a low publication bias, due no obvious asymmetries in Funnels plots for any study outcomes and Eggers Testing. • Large magnitude of effect: This outcome was judged to have a large magnitude of effect, as evidenced by an overall effect of p=0.008 (OR 0.72, 95%CI: 0.57 to 0.92).* | 246 per 1000 | 190 per 1000 | Difference: 56 fewer per 1000 patients<br>(95% CI: 89 to 15 fewer per 1000 patients)<br>Based on data from 4052 patients in 11 studies | OR 0.72<br>(0.57 to 0.92) | ⊕⊕⊕◯<br>MODERATE<br>Due to serious inconsistency. Due to serious imprecision. Upgraded due to large magnitude of effect. |

**Supplementary Table S1: Inclusion and exclusion criteria**

| | Title/Abstract | Screening | Full Text | Screening |
|---|---|---|---|---|
| | Inclusion criteria | Exclusion criteria | Inclusion criteria | Exclusion criteria |
| Study design | • Human studies.<br>• >50 cases. | • Animal studies.<br>• < 50 cases.<br>• Non-primary research articles (including reviews).<br>• Editorial comments, meeting abstracts (with insufficient data), book chapters, non-peer review articles. | • Human studies<br>• >50 cases<br>• Randomised controlled studies and prospective randomised controlled studies. | • Animal studies<br>• < 50 cases<br>• Non-primary research articles (including reviews).<br>• Editorial comments, meeting abstracts (with insufficient data), book chapters, non-peer review articles. |
| Group | • Adult men and women treated with extended release metformin compared to immediate release metformin | • Children | • Adult men and women treated with extended release metformin compared to immediate release metformin | • Children |
| Exposure | • Extended release metformin vs. immediate release metformin for any adult | • Metformin with other drug combinations. | • Extended release metformin vs. immediate release metformin for any adult | • Metformin with other drug combinations. |
| Outcome | • Glycaemic control Weight gain/loss<br>• Lipid profiles<br>• Side effects (including gastrointestinal)<br>• Cost-effectiveness<br>• Serum/tissue metformin levels (peak concentration/area under curve)<br>• Patient satisfaction<br>• Compliance with medication | | • Glycaemic control Weight gain/loss<br>• Lipid profiles<br>• Side effects (including gastrointestinal)<br>• Cost-effectiveness<br>• Serum/tissue metformin levels (peak concentration/area under curve)<br>• Patient satisfaction<br>• Compliance with medication | |

**Supplementary Table S2: Risk of Bias analysis**

| | Risk of Bias | | | | | | |
|---|---|---|---|---|---|---|---|
| Study or Subgroup | A | B | C | D | E | F | G |
| Aggarwal, 2018 | + | ? | + | + | + | + | + |
| Buse, 2016 | + | ? | + | + | ? | + | + |
| Castellotti, 2007 | ? | ? | ? | ? | − | + | + |
| Derosa, 2017 | ? | + | + | ? | + | + | + |
| Fineman, 2015 | ? | ? | ? | ? | ? | ? | + |
| Fujioka, 2003 | ? | ? | + | + | + | + | + |
| Gao, 2008 | ? | − | − | − | + | + | + |
| Hameed, 2017 | ? | ? | ? | ? | ? | + | + |
| Henry, 2018 | + | ? | + | ? | + | + | + |
| Hsieh, 2007 | ? | ? | + | + | + | + | + |
| Ji, 2018 | + | − | − | − | ? | + | + |
| Niecestro, 2003 | ? | ? | + | + | ? | + | + |
| Schwartz, 2006 | + | ? | + | + | + | + | + |
| Xue, 2012 | + | ? | ? | ? | ? | ? | + |
| Zheng, 2009 | ? | ? | − | − | + | + | + |

Supplementary Table S3: Study characteristics table

Please see separate supplementary Excel spreadsheet

S1 Text: PROSPERO document

NIHR | National Institute for Health Research

PROSPERO
International prospective register of systematic reviews

# Efficacy, acceptability, and side-effect profile of sustained versus immediate-release metformin formulations: a systematic review and meta-analysis

*Jane Tarry-Adkins, Imogen Grant, Susan Ozanne, Rebecca Reynolds, Catherine Aiken*

## Citation

Jane Tarry-Adkins, Imogen Grant, Susan Ozanne, Rebecca Reynolds, Catherine Aiken. Efficacy, acceptability, and side-effect profile of sustained versus immediate-release metformin formulations: a systematic review and meta-analysis. PROSPERO 2020 CRD42020194190 Available from: https://www.crd.york.ac.uk/prospero/display_record.php?ID=CRD42020194190

## Review question

Are outcomes for patients randomised to sustained-release formulations of metformin equivalent to outcomes for patients randomised to immediate-release metformin?

'Sustained-release' in this context will encompass slow release, extended release and delayed release preparations.

## Searches

PubMed: June 1997 to Sept 2020, Web of Science:1900 to Sept 2020, OVID EMBASE:1974 to Sept 2020, OVID MEDLINE: 1946 to Sept 2020, The Cochrane Database: Database inception to Sept 2020, Clintrials.gov: Database inception to Sept 2020.

No language, publication period or other filters will be used.

PubMed Search Criteria: Search date range: June 1997 to August 2020
Search: (metformin[MeSH Terms]) AND (delayed-action preparations[MeSH Terms]) Sort by: Most Recent

"metformin"[MeSH Terms] AND "delayed-action preparations"[MeSH Terms]

Translations

metformin[MeSH Terms]: "metformin"[MeSH Terms]

delayed-action preparations[MeSH Terms]: "delayed-action preparations"[MeSH Terms]

## Types of study to be included

Types of studies to be included

• Human studies.

• Randomised controlled and prospective randomised studies.

• Studies with 50 or more participants.

• Studies comparing sustained release formulations of metformin with immediate release metformin for any indication.

Exclusion criteria

• Animal studies.

• Studies randomising fewer than 50 participants.

• Studies including children.

• Non-randomised studies.

• Non primary research data (including reviews, book chapters, editorial comments and conference abstracts which contain insufficient data for analysis).

## Condition or domain being studied
Any conditions for which sustained release formulations of metformin and immediate release metformin have been trialled (including, but not limited to, type 2 diabetes and obesity).

## Participants/population
Participants who are randomised to receive extended release metformin or immediate release metformin. Both males and females adults will be included.

Types of studies to be included

• Human studies.

• Randomised controlled and prospective randomised studies.

• Studies with 50 or more participants.

• Studies comparing sustained release formulations of metformin with immediate release metformin for any indication.

Exclusion criteria

• Animal studies.

• Studies randomising fewer than 50 participants.

• Studies including children.

• Non-randomised studies.

• Non primary research data (including reviews, book chapters, editorial comments and conference abstracts which contain insufficient data for analysis).

## Intervention(s), exposure(s)
Sustained release formulations of metformin.

## Comparator(s)/control
The reference group will be immediate release metformin.

## Main outcome(s)
Primary outcomes

• Glycaemic control (including, but not limited to fasting blood glucose, random blood glucose, Hb1Ac, need for additional therapy, time spent in target blood glucose range)

• Weight gain/loss

• Lipid profiles

• Side effects (including gastrointestinal)

## * Measures of effect

The principle summary measures utilised in this systematic review will be unadjusted odds ratios (for dichotomous data) or differences in means (for continuous data).

## Additional outcome(s)

Secondary outcomes

• Cost-effectiveness

• Serum/tissue metformin levels (peak concentration/area under curve)

• Patient satisfaction

• Compliance with medication

## * Measures of effect

The principle summary measures utilised in this systematic review will be unadjusted odds ratios (for dichotomous data) or differences in means (for continuous data).

## Data extraction (selection and coding)

PubMed, Ovid Embase, MEDLINE, Web of Science, The Cochrane Library and clintrials.gov will be searched systematically, after which the papers will be screened on Title and Abstract, by two reviewers independently. The full texts of these selected studies will be independently assessed using inclusion and exclusion criteria. Disagreement over the eligibility will be discussed with a third reviewer.

We intend to extract the following data: author, year of publication, country, sample size, exposure unit (mg), duration of exposure to metformin, diagnostic criteria for type 2 diabetes or other indications, population randomisation criteria, reported outcomes including, but not limited to baseline characteristics, side effects, drug preference and treatment compliance, glycaemic control, weight and lipid profiles, metformin pharmacokinetic/pharmacodynamics data.

## Risk of bias (quality) assessment

The quality of studies will be assessed using the modified Cochrane Collaboration tool to assess risk of bias for randomized controlled trials. Bias is assessed as a judgment (high, low, or unclear) for individual elements from seven domains: (selection (randomisation), selection (concealment), performance, detection, attrition, reporting, and other). This assessment will be performed by two reviewers independently. Disagreement between reviewers regarding the quality of a study will be discussed with a third reviewer.

## Strategy for data synthesis

To synthesise and analyse quantitative data, a systematic review/meta-analysis will be conducted using R. Heterogeneity will be assessed with Galbraith plots, and the decision to use a fixed-effect or random-effects model will based on this analysis. Data will be graphically displayed using forest plots. Additionally, meta-regression will be performed to explore the effects of heterogeneity in terms of study-level covariates. Publication bias will be assessed using funnel plots, plotting the effects sizes against standard errors.

## Analysis of subgroups or subsets

If the number of studies allow, sub-grouping analysis indication, formulation, geographical area of the study, or dose given may be conducted.

## Contact details for further information

Jane Tarry-Adkins
janeadkins@googlemail.com

## Organisational affiliation of the review

University of Cambridge

## Review team members and their organisational affiliations

Mrs Jane Tarry-Adkins. University of Cambridge
Miss Imogen Grant. University of Cambridge

Professor Susan Ozanne. University of Cambridge
Professor Rebecca Reynolds. University of Edinburgh
Dr Catherine Aiken. University of Cambridge

## Type and method of review
Intervention, Meta-analysis, Systematic review

## Anticipated or actual start date
03 August 2020

## Anticipated completion date
07 December 2020

## Funding sources/sponsors
The British Heart Foundation and the Medical Research Council.

## Grant number(s)

State the funder, grant or award number and the date of award

The British Heart Foundation: RG/17/12/33167, the Medical Research Council:MC_UU_00014/4.

## Conflicts of interest

## Language
English

## Country
England

## Stage of review
Review Ongoing

## Subject index terms status
Subject indexing assigned by CRD

## Subject index terms
Diabetes Mellitus, Type 2; Drug-Related Side Effects and Adverse Reactions; Humans; Hypoglycemic Agents; Metformin

## Date of registration in PROSPERO
13 July 2020

## Date of first submission
25 June 2020

## Stage of review at time of this submission

| Stage | Started | Completed |
|---|---|---|
| Preliminary searches | Yes | No |
| Piloting of the study selection process | No | No |
| Formal screening of search results against eligibility criteria | No | No |
| Data extraction | No | No |
| Risk of bias (quality) assessment | No | No |
| Data analysis | No | No |

## Revision note

Two reviewers have been added onto this review and wehave clarified our intervention group in more detail.

*The record owner confirms that the information they have supplied for this submission is accurate and complete and they understand that deliberate provision of inaccurate information or omission of data may be construed as scientific misconduct.*

*The record owner confirms that they will update the status of the review when it is completed and will add publication details in due course.*

## Versions

13 July 2020
24 September 2020

PROSPERO
This information has been provided by the named contact for this review. CRD has accepted this information in good faith and registered the review in PROSPERO. The registrant confirms that the information supplied for this submission is accurate and complete. CRD bears no responsibility or liability for the content of this registration record, any associated files or external websites.

**S2 text: Database search terms**

**<u>PubMed: June 1997 to 27th November 2020</u>**

Search: (metformin[MeSH Terms]) AND (delayed-action preparations[MeSH Terms]) Sort by: Publication Date

"metformin"[MeSH Terms] AND "delayed-action preparations"[MeSH Terms]

Translations

metformin[MeSH Terms]: "metformin"[MeSH Terms]

delayed-action preparations[MeSH Terms]: "delayed-action preparations"[MeSH Terms]

**<u>Web of Science: 1900 to 27th November 2020</u>**

TI=(metformin* AND extended release) OR

TI=(metformin* AND sustained release) OR

TI=(metformin* AND controlled release) OR

TI=(metformin* AND slow release) OR

TI=(metformin* AND timed release)

**<u>OVID EMBASE: 1974 to 27th November 2020</u>**

(metformin and extended release).mp. OR

(metformin and sustained release).mp. OR

(metformin and controlled release).mp. OR

(metformin and slow release).mp. OR

(metformin and timed release).mp.

**<u>OVID MEDLINE: 1946 to 27th November 2020</u>**

(metformin and extended release).mp. OR

(metformin and sustained release).mp. OR

(metformin and controlled release).mp. OR

(metformin and slow release).mp. OR

(metformin and timed release).mp.

**<u>Cochrane Library: Database inception to 27th November 2020</u>**

metformin AND delayed-action preparations

**<u>Clinicaltrial.gov: Database inception to 27th November 2020</u>**

metformin AND delayed-action preparation
